# Surface immobilization of single atoms on heteroatom-doped carbon nanospheres through phenolic-mediated interfacial anchoring for highly efficient biocatalysis†

**DOI:** 10.1039/d4sc07775j

**Published:** 2025-01-20

**Authors:** Yajing Zhang, Yunxiang He, Yun Jiao, Guobin Yang, Yiran Pu, Zhangmin Wan, Shuyun Li, Yanchao Wu, Wen Liao, Junling Guo

**Affiliations:** a BMI Center for Biomass Materials and Nanointerfaces, National Engineering Laboratory for Clean Technology of Leather Manufacture, Ministry of Education Key Laboratory of Leather Chemistry and Engineering, College of Biomass Science and Engineering, Sichuan University Chengdu Sichuan 610065 China junling.guo@scu.edu.cn; b Weihai Marine Organism & Medical Technology Research Institute, College of Marine Science and Technology, Harbin Institute of Technology Weihai Shandong 264209 China; c Bioproducts Institute, Department of Chemical and Biological Engineering, The University of British Columbia Vancouver BC V6T 1Z4 Canada junling.guo@ubc.ca; d State Key Laboratory of Oral Diseases & National Clinical Research Center for Oral Diseases, West China Hospital of Stomatology, Sichuan University Chengdu Sichuan 610041 China; e State Key Laboratory of Polymer Materials Engineering, Sichuan University Chengdu Sichuan 610065 China

## Abstract

Single-atom catalysts (SACs) dispersed on support materials exhibit exceptional catalytic properties that can be fine-tuned through interactions between the single atoms and the support. However, selectively controlling the spatial location of single metal atoms while simultaneously harmonizing their coordination environment remains a significant challenge. Here, we present a phenolic-mediated interfacial anchoring (PIA) strategy to prepare SACs with Fe single atoms anchored on the surface of heteroatom-doped carbon nanospheres. Briefly, by exploiting metal-phenolic networks (MPNs) for surface coating and phloroglucinol-induced polymerization for support precursor formation, we successfully anchored Fe single atoms at the interface between the MPN layer and the support surface. Moreover, this anchoring strategy effectively prevents Fe species from clustering or migrating toward the interior of the support during thermal treatment, resulting in atomically dispersed FeN_3_P-SAC that exhibits a high metallic utilization efficiency and comparable peroxidase-like catalytic activity and kinetics to natural enzymes. As a proof-of-concept demonstration, FeN_3_P-SAC could effectively block the growth of tumor cells *in vitro* by combining excellent tumor penetration and the ability to activate chemodynamic and photothermal effects synergistically. This work advances the development of highly active SACs with MPN-based nanotechnology, providing a promising approach for nanocatalytic tumor therapy.

## Abstract 2

Single-atom catalysts (SACs) *via* phenolic-mediated interfacial anchoring (PIA) with single Fe atoms dispersed on the surface of N/P-doped carbon supports, exhibiting exceptional biocatalytic properties.

## Graphical abstract

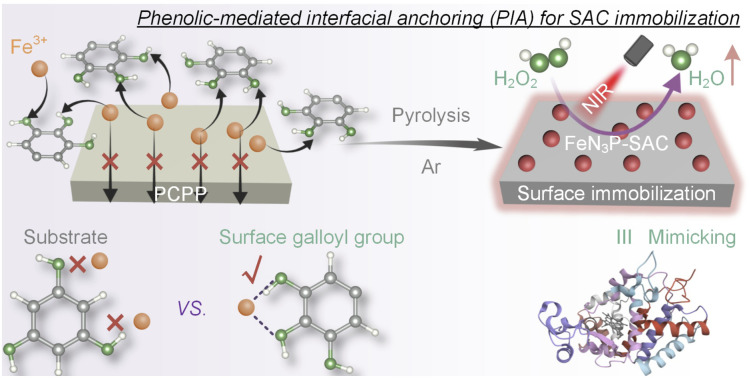

## Introduction

**Fig. 1:**
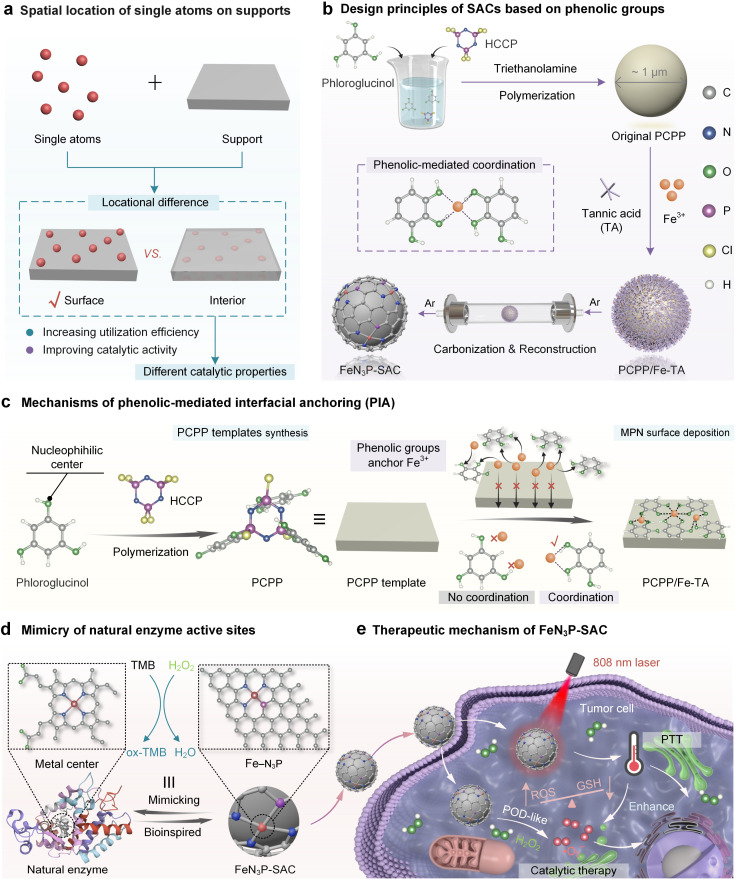
Surface immobilization of single atoms on heteroatom-doped carbon through phenolic-mediated interfacial anchoring for highly efficient biocatalysis. (a) Schematic of the spatial location of single atoms on supports. (b) Design principles of SACs based on phenolic groups. (c) Mechanisms of phenolic-mediated interfacial anchoring (PIA). (d) Mimicry of natural enzyme active sites. (e) Schematic of the therapeutic mechanism of FeN_3_P-SAC in nanocatalytic therapy.

Biological enzymes use specific metal ions as active sites to catalyze various biochemical reactions in organisms under mild conditions.^1–4^ Owing to their fragile nature, low stability, high cost, and difficult storage, the wide industrial utilization of natural enzymes has been limited.^5–7^ Developing artificial enzymes with better catalytic performance and enhanced stabilities over natural enzymes has been a long-standing goal in catalysis. Single-atom catalysts (SACs) as a newly emerged class of artificial enzymes have attracted increasing attention in recent years, which integrated state-of-the-art single-atom technology with intrinsic enzyme-like active sites.^5^ Among these artificial enzymes, metal–nitrogen–carbon (M–N–C, M = Fe, Co, Zn, Cu, Ni, *etc.*) materials with isolated metal atoms anchored onto a carbon support through the nitrogen have been extensively investigated due to their preferable peroxidase-like characteristics.^8–10^ In particular, Fe-based SACs are recognized as the most promising enzymes as they closely mimic the Fe-active centers of natural horseradish peroxidase (HRP) and exhibit exceptional catalytic activity.^11,12^ Apart from the central metals, the catalytic performance of SACs is highly dependent on the interactions between the single atoms and the support material. The factors regulating the local environment surrounding each single atom, including coordination species, coordination number, spatial microenvironment, and spatial location, are crucial for SAC design.^7,13,14^ Conventional fabrication methods usually lead to locational differences of single atoms on supports, either attaching to the surface by adsorption or being embedded within the interior of the support (Fig. 1a), as the SAC precursor is usually a matrix prepared by mixing metallic species with the organic support precursors, resulting in the encapsulation of metallic centers within the matrix. The surface single atoms are fully exposed with a high utilization efficiency, while the interior embedded single atoms are blocked by the dense graphitic domains or solvent flooding in micropores and remain inactive.^15–19^ Particularly, these internally inaccessible atomic species cause the waste of metallic resources and the uneven and unstable distribution of the single metal atoms.

Recently, surface-coating strategies have been developed to achieve the surface immobilization of SACs by using silica shells, crosslinked polymer layers, and metal-phenolic networks (MPNs).^16,20–25^ Although the above synthesis techniques have been well studied, there are still some operational considerations. For example, the removal of silica shells often involves the use of hazardous solvents such as sodium hydroxide and hydrofluoric acid, raising a variety of safety concerns.^16^ Monomers forming polymers are not only typically restricted to a handful of choices but also need to be determined on a case-by-case basis.^21,22^ Encouragingly, MPNs can be rapidly deposited on a broad range of supports or interfaces regardless of their structures and shapes based on the universal adhesive properties of natural polyphenols.^26–29^ However, owing to the lack of heteroatom elements (*e.g.*, B, O, N, S, P, and F) in polyphenols, metal ions in MPNs cannot be efficiently and steadily converted into single atoms at supports.^22,30–34^ The incorporation of exogenous heteroatoms (*e.g.*, NH_3_) can assist the fabrication of SACs from polyphenols while making it challenging to uniformly or accurately arrange diverse heteroatoms for anchoring single metal atoms at the interface.^22^ Thus, seeking an effective synthesis strategy for SACs that achieves both surface loading of single atoms and coordination environments enriched with multiple heteroatoms is still in high demand.

Here, we report a phenolic-mediated interfacial anchoring (PIA) strategy to achieve the outermost surface immobilization of single atoms on heteroatom-doped carbon nanospheres for highly efficient biocatalysis. This was achieved by first coating an N/P-doped poly(cyclotriphosphazene (HCCP)-*co*-phloroglucinol) template (PCPP) with a thin MPN film (Fig. 1b). In this process, the Fe^3+^ ions were uniformly introduced on PCPP surfaces by phenolic groups in tannic acid (TA) based on a PIA strategy. More importantly, phloroglucinol and HCCP in PCPP cannot coordinate with Fe^3+^ ions to prevent the inward migration of metal ions and ensure the high utilization efficiency of metallic sources (Fig. 1c). Based on the mechanisms above, the Fe species only immobilizes on the outermost surface of carbon nanospheres, leaving the active site completely exposed (FeN_3_P-SAC) under one-step pyrolysis in an argon (Ar) atmosphere. Furthermore, structural characterization demonstrated that Fe atoms were atomically dispersed in FeN_3_P-SAC with well-defined Fe-N_3_P sites, which were similar to the active centers of natural enzymes (Fig. 1d). We also demonstrate that FeN_3_P-SAC shows comparable catalytic activity and kinetics to the natural enzyme peroxidase. Beyond catalytic function, the photothermal effect achieved by FeN_3_P-SAC can additionally help enhance the effectiveness of enzyme catalytic activity, thereby achieving efficient nanocatalytic therapy (Fig. 1e).

## Results and discussion

### Synthesis and characterization of PCPP/Fe-TA

**Fig. 2:**
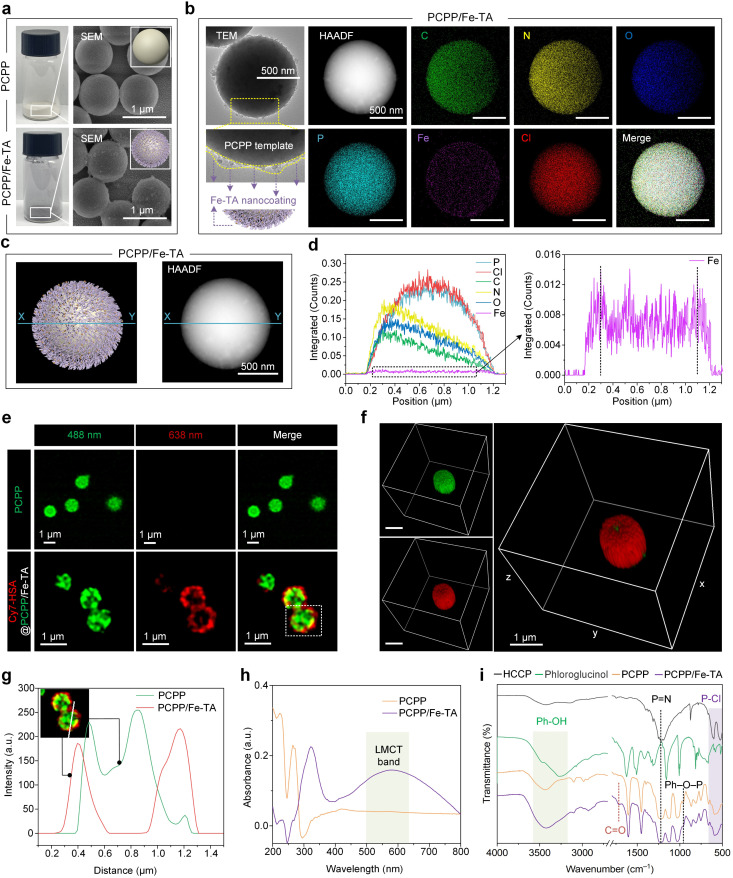
Surface deposition of MPNs on PCPP templates based on the PIA strategy. (a) Optical photographs and SEM images of PCPP and PCPP/Fe-TA powders. The inset shows the 3D model diagram of PCPP (top) and PCPP/Fe-TA (bottom) powders. Scale bars, 1 μm. (b) TEM images, HAADF images, and the corresponding EDX elemental mapping images of PCPP/Fe-TA powders. Scale bars, 500 nm. The yellow lines in the enlarged TEM image indicate a rough Fe-TA nanocoating. The 3D model diagram of PCPP/Fe-TA further shows the Fe-TA nanocoating. (c) 3D model diagram (left) and HAADF image (right) with the blue line X–Y for PCPP/Fe-TA powders. (d) EDX line scanning profiles (left) and enlarged view (right) along the blue line X–Y in (c) of PCPP/Fe-TA powders. (e) CLSM images of PCPP templates (green) and PCPP/Fe-TA labeled with Cy7-HSA (red). (f) 3D CLSM images of PCPP/Fe-TA labeled with Cy7-HSA (red). Scale bars, 1 μm. (g) Fluorescence colocalization analysis of the selected region in (e). (h) UV-Vis spectra of PCPP/Fe-TA powder. (i) FT-IR spectra of the free monomers (HCCP and phloroglucinol), PCPP template, and PCPP/Fe-TA.

The synthesis of PCPP/Fe-TA is schematically illustrated in Fig. 1b. The spherical hydrophobic PCPP with a smooth surface as the core template was first prepared by a polycondensation reaction that can simultaneously introduce a high load of heteroatoms N and P (Fig. S1†). Subsequently, Fe^3+^ ions were anchored on the surface of PCPP *via* phenolic-mediated strong coordination to obtain a PCPP/Fe-TA core–shell composite with rough surfaces. The changes in powder color, zeta potential measurements, and dynamic light scattering (DLS) preliminarily confirmed the formation of the Fe-TA nanocoating (Fig. 2a, left; Fig. S2 and S3†). Scanning electron microscopy (SEM), transmission electron microscopy (TEM), and high-angle annular dark-field scanning TEM (HAADF-STEM) images indicated that PCPP/Fe-TA retained a similar spherical morphology to the original PCPP with a rough surface (Fig. 2a and b, left). Energy dispersive X-ray spectroscopy (EDX) showed that C, N, O, P, and Cl elements were distributed in the core, while the Fe signal was mostly only found in the shell (Fig. 2b, right). In addition, EDX line scanning profiles provided additional support for the distribution of Fe^3+^ ions on the surface of the PCPP (Fig. 2c and d). Notably, confocal laser scanning microscopy (CLSM) images and 3D CLSM images also further confirmed the attachment of Fe^3+^ ions on the surfaces of the PCPP, in which the Fe-TA nanocoating (red fluorescence) labeled with cyanine7-human serum albumin (Cy7-HSA) was not co-located with PCPP (green fluorescence) (Fig. 2e–g, S4 and S5†).

The chemical structures and composition of the PCPP and PCPP/Fe-TA have been further confirmed by UV-Vis absorption spectra, Fourier transform infrared spectrophotometry (FT-IR), and X-ray photoelectron spectroscopy (XPS). As compared to the PCPP, the appearance of the characteristic ligand-to-metal charge-transfer (LMCT) band in PCPP/Fe-TA, with a peak around 570 nm wavelength, suggested the existence of Fe-TA coordination (Fig. 2h).^35^ FT-IR spectra of comonomers (HCCP and phloroglucinol), as-synthesized PCPP, and PCPP/Fe-TA demonstrate the successful polymerization of comonomers (Fig. 2i). In the spectra of PCPP, the appearance of the Ph–O–P peak (958 cm^−1^) and the decrease in the intensity of the P–Cl peak (530 cm^−1^ and 615 cm^−1^) inferred complete polymerization.^36,37^ In addition, all characteristic peaks for PCPP could be observed in the spectra of PCPP/Fe-TA. The distinct peak at 1717 cm^−1^ can be attributed to the C=O groups, which further identified the presence of TA. XPS survey spectra of PCPP/Fe-TA demonstrated the elemental distribution of C, N, O, P, Cl, and a trace of Fe similar to EDX analysis (Fig. S6a†). High-resolution C 1s, O 1s, N 1s, P 2p, and Fe 2p spectra of PCPP/Fe-TA also indicated the polycondensation of co-monomers as well as successful capture of Fe^3+^ ions (Fig. S6b–f†), being consistent with that of the FT-IR observation.

Moreover, XRD measurements were carried out to evaluate the transformation of the crystallographic structure during the polycondensation process and before and after the Fe-TA nanocoating. A broad peak at 2*θ* = 20°–30° was observed in PCPP and PCPP/Fe-TA compared to comonomers, indicating the formation of amorphous microspheres (Fig. S7†). The specific surface area of PCPP decreased from 7.78 m^2^ g^−1^ to 6.92 m^2^ g^−1^ for PCPP/Fe-TA according to Brunauer–Emmett–Teller (BET) specific surface area analysis (Fig. S3 and S8†).

### Synthesis and structural characterization of FeN_3_P-SAC

To obtain the SACs *via* carbonization and gas-migration strategies, the mass loss of PCPP/Fe-TA was first investigated using thermogravimetric experiments (Fig. S9†). Predictably, PCPP and PCPP/Fe-TA exhibited superior thermal stability compared with TA. When the specimen was heated up to 800 °C, the char yields of the PCPP and PCPP/Fe-TA were over 60%. Subsequently, the as-obtained PCPP/Fe-TA was further pyrolyzed under a flowing Ar atmosphere to obtain FeN_3_P-SAC with an Fe-N/P-C structure, where the Fe-TA nanocoating served as the Fe source and PCPP served as the precursor of N/P-doped carbon. As a control, PCPP-800 without the Fe-TA nanocoating was also prepared by the same method. During pyrolysis, Fe^3+^ ions from the Fe-TA nanocoating diffused inward into the spherical structure, while N and P atoms from PCPP were released outward.^22,24^ Notably, due to the interfacial anchoring of the phenolic groups in TA with the Fe^3+^ ions from the surface shell, the inward release is lower than the outward diffusion, leading to Fe species being retained on the surface of the N/P-doped carbon support.^19^ Meanwhile, PCPP with covalent bonds and TA with a relatively large molecular weight (*M*_w_ ≈ 1700 Da) can induce the formation of a carbon support and an ordered rigid shell, preventing the collapse of spherical morphology.

**Fig. 3:**
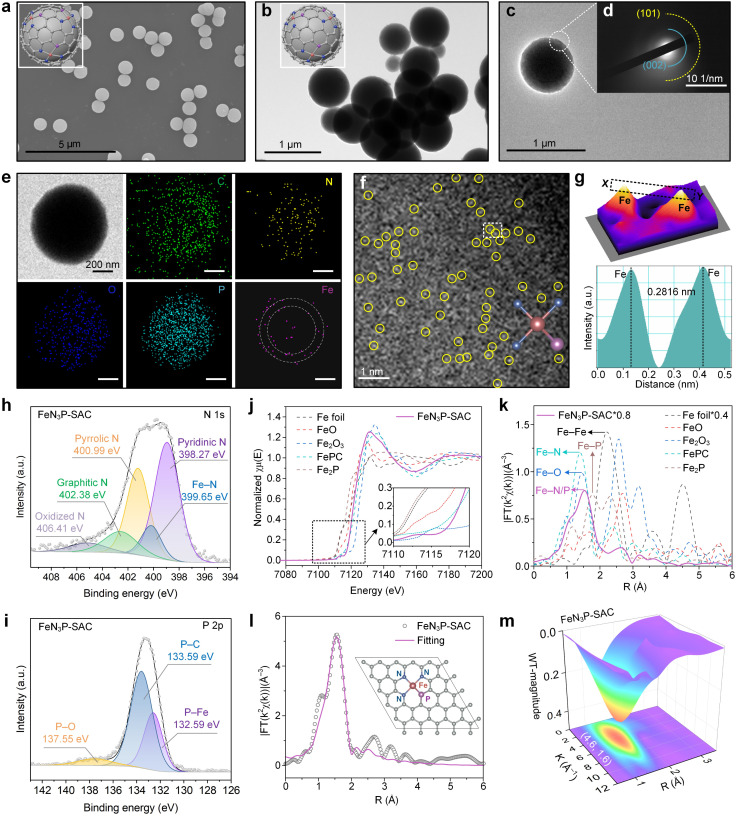
Morphology and structural characterization of FeN_3_P-SAC. (a) SEM image and (b) TEM image of FeN_3_P-SAC. Scale bars, 5 μm and 1 μm. (c) HRTEM image and (d) SAED pattern of FeN_3_P-SAC. Scale bar, 1 μm. (e) TEM image and the corresponding EDX elemental mapping images of FeN_3_P-SAC. The white dashed circles indicate the distribution of Fe element within FeN_3_P-SAC. Scale bars, 200 nm. (f) AC HAADF-STEM image of FeN_3_P-SAC. Scale bars, 1 μm. (g) 3D topographic atom image of the white dashed area in (f) and corresponding atomic intensity profiles (bottom) along the line X–Y in (g). (h and i) High-resolution XPS spectra of N 1s (h) and P 2p (i) for FeN_3_P-SAC. (j) Fe K-edge XANES spectra for FeN_3_P-SAC and reference samples. (k) FT-EXAFS based on the Fe K-edge XANES spectra for FeN_3_P-SAC and reference samples in *R* space. (l) Fitting result and corresponding structural model of FeN_3_P-SAC. (m) WT EXAFS contour plots of the Fe K-edge for FeN_3_P-SAC.

The SEM and TEM images indicated that FeN_3_P-SAC retained the initial spherical morphology with a rough surface (Fig. 3a–c). Selected-area electron-diffraction (SAED) analysis only showed a ring-like pattern with visible (002) and (101) lattice planes, tentatively validating the graphitic structure of the obtained carbon matrix without any highly crystallized phases (Fig. 3d), which is in line with previously reported carbon-based SACs. Notably, EDX analysis showed that C, N, O, and P elements were homogeneously dispersed throughout the entire architecture of FeN_3_P-SAC, in which Fe signals were mainly concentrated in the shell (white dashed circles) (Fig. 3e). Corresponding to SAED analysis, the XRD patterns of PCPP-800 and FeN_3_P-SAC showed only two broad peaks located near 25° and 44°, which were attributed to the characteristic diffractions of graphitic carbon (Fig. S10†). No signals of Fe nanoparticles were detected, indicating no obvious aggregation or clusters of the Fe species formed. Raman spectra were further used to evaluate their graphitization degrees through the intensity ratios of D and G bands (*I*_D_/*I*_G_) (Fig. S11†). Compared with PPCP-800 (1.10), the higher *I*_D_/*I*_G_ value of FeN_3_P-SAC (1.31) indicated that Fe doping generates more defective sites.^38^ Moreover, the 2D band peak of FeN_3_P-SAC is weaker compared to that of PCPP-800, indicating that Fe single atoms in FeN_3_P-SAC enhance the carbon disorder and reduce the graphitization degree, consistent with the *I*_D_/*I*_G_ analysis.^39^ Particularly, N_2_-sorption isotherms are recorded to evaluate the textural information of the framework, confirming a type-I sorption isotherm and mesoporous structures of FeN_3_P-SAC. Among them, the FeN_3_P-SAC sample exhibits a large BET surface area (321.6789 m^2^ g^−1^) with rich mesopores (<2 nm) (Fig. S12†). In addition, aberration-corrected high-angle annular dark-field scanning transmission electron microscopy (AC HAADF-STEM) was further performed to examine FeN_3_P-SAC at the atomic scale. As shown in Fig. 3f, the obvious individual bright dots (highlighted by yellow circles) showed the atomic dispersion of single Fe atoms over FeN_3_P-SAC. The average diameter of these bright dots was measured to be 0.2816 nm, which is substantially larger than the effective diameter of the Fe atom, further validating the atomically dispersed Fe atoms on the support (Fig. 3g).

XPS and element-selective X-ray absorption spectroscopy (XAS) were employed to disclose the surface electronic states and the coordination environment of FeN_3_P-SAC. The XPS survey spectra manifested the presence of C, O, N, P, and Fe elements in all samples, in which the C 1s spectra showed four forms of C species and the O 1s spectra exhibited three types of O species (Fig. S13a–c†). The Fe 2p spectra with two relatively weak peaks centered at 712.9 eV (Fe 2p_3/2_) and 723.3 eV (Fe 2p_1/2_) suggested the positive oxidation states of Fe species in FeN_3_P-SAC (Fig. S13d†). Notably, the N 1s spectra displayed five deconvoluted components at 398.27 (pyridinic N), 399.65 (Fe–N), 400.99 (pyrrolic N), 402.38 (graphitic N), and 406.41 eV (oxidized N) (Fig. 3h). Most importantly, the characteristic peaks at 132.59 eV, corresponding to the Fe–P bond, were also observed in the P 2p spectra for FeN_3_P-SAC, which could stem from the partial replacement of N atoms with P to form the Fe–P bond (Fig. 3i). Meanwhile, the P 2p spectra exhibited two broad peaks located at 133.59 and 137.855 eV, which could be assigned to P–C and P–O bonds, respectively.^6,34^ These results indicated that the P and N species were doped into the carbon framework with typical Fe–N and Fe–P dual-coordinating environments. Inductively coupled plasma optical emission spectrometry (ICP-OES) was further applied to quantify the Fe content, which is 1.75 wt% for FeN_3_P-SAC (Fig. S14†).

Furthermore, the coordination environment between Fe and N/P was determined by X-ray absorption energy near-edge structure (XANES) and Fourier transform extended X-ray absorption fine structure (FT-EXAFS) measurements. The Fe K-edge XANES spectra demonstrated that the line position (the enlarged view of the pre-peak) of FeN_3_P-SAC was located between Fe foil and Fe_2_O_3_, indicating a positive valence state between 0 and +3, in line with the deduction from high-resolution Fe 2p spectra (Fig. 3j). Notably, the threshold position of FeN_3_P-SAC was close to that of FePc, suggesting this catalyst contains a similar FeN_4_ structure. Additionally, FT-EXAFS showed a relatively broad peak at around 1.53 Å, which could be ascribed to the coexistence of Fe–N (1.41 Å) and Fe–P (1.77 Å) coordination (Fig. 3k).^34,40^ In contrast to the Fe foil and Fe-containing control materials (FeO, Fe_2_O_3_, and Fe_2_P), no Fe–Fe peaks at 2.2 Å or larger bond distances were detected, manifesting atomically dispersed Fe–H configuration. Further fitting curves in the *R* and *K* spaces indicated the coordination configuration of Fe moieties in FeN_3_P-SAC (Fig. 3l, S15, and S16†). The best-fitting analyses for FeN_3_P-SAC showed Fe–N with a coordination number of 3.2 and Fe–P with a coordination number of 0.7, confirming the asymmetric four-coordinated Fe–N_3_P_1_ structure (Table S1†). Besides, the atomic structure model of FeN_3_P-SAC was successfully constructed based on the fitting results (inset of Fig. 3l). These results were further supported by the wavelet transform (WT) analysis of the *k*^2^-weighted EXAFS spectra (Fig. 3m and S17†). Different from the WT maximum peak of Fe foil (8.0 Å^−1^) and Fe_2_P, the WT maximum peak of FeN_3_P-SAC exhibited one intensity maximum at approximately 4.6 Å^−1^, which is close to that in FePc. Consequently, the Fe species in FeN_3_P-SAC were confirmed to be atomically distributed in an FePc-like coordination environment.

### Peroxidase-like activity characterization of FeN_3_P-SAC

**Fig. 4:**
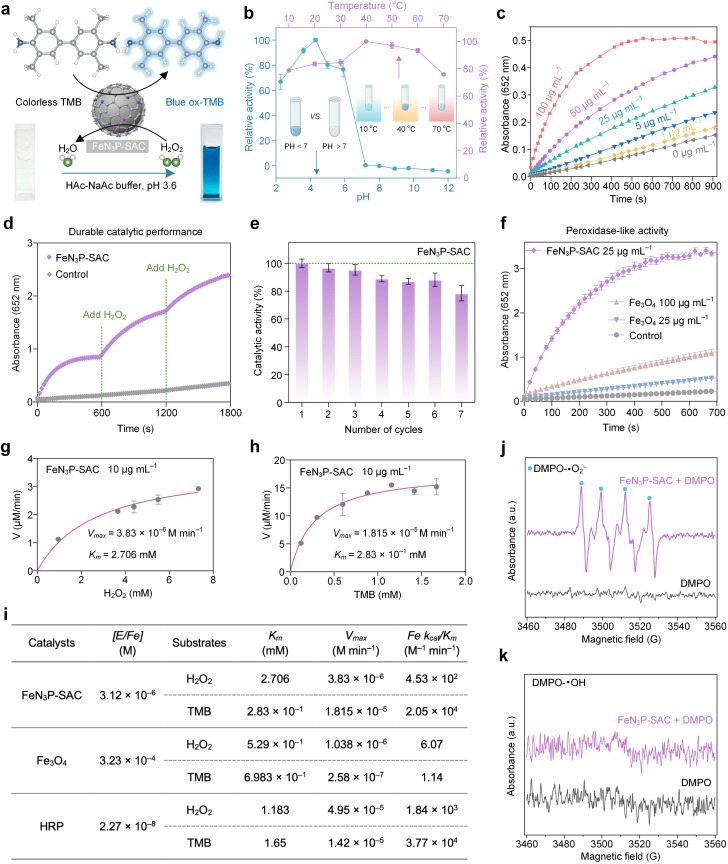
Natural enzyme-like properties of FeN_3_P-SAC. (a) FeN_3_P-SAC shows POD-like activity by catalyzing the oxidation of peroxidase substrates (TMB) to enable colorimetric reactions. (b) Effects of pH and temperature on the catalytic activity of FeN_3_P-SAC. (c) TMB chromogenic curves for varied concentrations of FeN_3_P-SAC over time in HAc–NaAc buffer (pH 3.6) under H_2_O_2_ (0.915 mM) and TMB (0.064 mM). (d) TMB chromogenic curves for FeN_3_P-SAC (55.56 μg mL^−1^) upon the addition of H_2_O_2_ at 600 s, 1200 s, and 1800 s in HAc–NaAc buffer (pH 3.6) under H_2_O_2_ (0.867 mM) and TMB (0.275 mM). The control indicates no addition of samples. (e) Catalytic recyclability of FeN_3_P-SAC (50 μg mL^−1^) in the presence of H_2_O_2_ (1.462 mM) and TMB (0.623 mM). (f) Reaction-time curves of the TMB colorimetric reaction catalyzed by FeN_3_P-SAC (25 μg mL^−1^), Fe_3_O_4_ (25 and 100 μg mL^−1^), and the control under H_2_O_2_ (14.625 mM) and TMB (0.465 mM). The control indicates no addition of samples. (g and h) Characterization of the catalytic kinetics by FeN_3_P-SAC. The initial reaction velocity (v) was measured in HAc–NaAc buffer (pH 3.6). When the concentration of H_2_O_2_ was varied, the concentration of TMB used for FeN_3_P-SAC was 0.622 × 10^−3^ M. When the concentration of TMB varied, the concentration of H_2_O_2_ was 0.141 M. (i) Comparison of the kinetics activity based on Fe active sites doped on FeN_3_P-SAC, Fe_3_O_4_, and natural HRP. ESR spectra of radicals trapped by DMPO in DMSO solution (j) or water solution (k) with FeN_3_P-SAC. For (b), (e), (f), and (g and h), *n* = 3 independent measurements, with data presented as means ± s.d.

The P species, an essential component of many natural enzymes, could enhance the peroxidase-like (POD-like) activity of the Fe center through long-range interactions.^41–43^ Based on the Fe-N_3_P active sites in FeN_3_P-SAC, we tested the POD-like activities of FeN_3_P-SAC by using 3,3′,5,5′-tetramethylbenzidine (TMB) as the substrate and hydrogen peroxide (H_2_O_2_) as the oxidant, in which colorless TMB was catalyzed to produce blue-colored oxidized TMB (ox-TMB) (Fig. 4a). Compared to PCPP-800 without Fe species, FeN_3_P-SAC exhibited markedly higher POD-like activity, indicating an efficient single-atom peroxidase mimic and that Fe species mainly act in a similar way to POD (Fig. S18 and S19†). In addition, the higher catalytic activity of FeN_3_P-SAC was observed over a wide range of temperatures (30–60 °C) and pH values (3.0–5.0) (Fig. 4b). To make a valid comparison between FeN_3_P-SAC and natural horseradish peroxidase (HRP), all post-measurements were performed at an identical temperature of 37 °C and a pH of 3.6.^6,44^ Except for temperatures and pH, the POD-like catalytic performance also strongly depends on the reaction time and concentrations. The absorbance at 652 nm increased with reaction time and the FeN_3_P-SAC concentration, indicating the continuous generation of reactive oxygen species (ROS) *via* POD-like activities (Fig. 4c). In particular, along with the constant H_2_O_2_ supplementation, FeN_3_P-SAC also exhibited sustained catalytic generation of ROS, maintaining high catalytic activity even after 0.5 h of reaction (Fig. 4d). More importantly, FeN_3_P-SAC has also been demonstrated to sustain biocatalytic function and retain over 80% of its bioactivity even after 7 reaction cycles (Fig. 4e). Given the possibility of metal dissolution in SACs, real-time monitoring of Fe leaching from FeN_3_P-SAC in the catalytic reaction solution is necessary.^45^ Notably, the corresponding Fe leaching amount was still very low and almost close to the detection limit even when the catalysts underwent 24 h of HAc–NaAc buffer solution (acidic, pH 3.6) treatment (Fig. S20a†).

Furthermore, the treated catalysts can still retain at least 92% of the POD-like activity, underscoring their remarkable catalytic performance and exceptional durability (Fig. S20b and c†). Based on the above analysis, FeN_3_P-SAC showed much higher catalytic activity compared to the most widely explored commercial Fe_3_O_4_ nanozyme (Fig. 4f).

To further evaluate the catalytic performance of FeN_3_P-SAC, we carried out steady-state kinetic measurements. Two other Fe-based nanozymes, including commercial Fe_3_O_4_ nanozyme and natural HRP, were used as control samples to investigate the catalytic efficiency of various Fe active sites (Fig. 4g, h, and S21†). All the kinetic parameters of these nanozymes are presented in Fig. 4i. Fe single atoms within FeN_3_P-SAC possessed a comparable catalytic efficiency (*K*_cat_/*K*_m_) to H_2_O_2_ (4.53 × 10^2^ M^−1^ min^−1^*vs.* 1.84 × 10^3^ M^−1^ min^−1^) and TMB (2.05 × 10^4^ M^−1^ min^−1^*vs.* 3.77 × 10^4^ M^−1^ min^−1^) with natural HRP. In addition, when H_2_O_2_ acted as the substrate and TMB concentration was fixed, the catalytic efficiency of FeN_3_P-SAC was 7.46 × 10^1^ times higher than that of the commercial Fe_3_O_4_ nanozyme. Meanwhile, when TMB acted as a reaction substrate and H_2_O_2_ concentration was fixed, the catalytic efficiency of FeN_3_P-SAC was 1.79 × 10^4^ times higher than that of the commercial Fe_3_O_4_ nanozyme. These results provided strong evidence that artificial nanozymes, such as FeN_3_P-SAC, catalyzing enzyme-like reactions can possess the fundamental properties of natural enzymes, differing in that FeN_3_P-SAC with Fe-N_3_P sites shows promising potential to replace natural enzymes by engineering the coordination environment of the metal active center.

Furthermore, to reveal the catalytic reaction mechanism of FeN_3_P-SAC, electron spin resonance (ESR) spectroscopy was used to examine the ROS intermediates being produced during the catalytic reaction. We observed the formation of a superoxide radical (·O_2_^−^) during the activation of H_2_O_2_ by FeN_3_P-SAC with 5,5-dimethyl-1-pyrroline *N*-oxide (DMPO) as a spin-trapping agent (Fig. 4j). In contrast, the signal intensity of the hydroxyl radical (·OH) was negligible during the activation of H_2_O_2_ by FeN_3_P-SAC, confirming that ·O_2_^−^ is the major POD-like catalytic product of FeN_3_P-SAC (Fig. 4k).

### FeN_3_P-SAC inhibits tumor cell growth *in vitro*

**Fig. 5:**
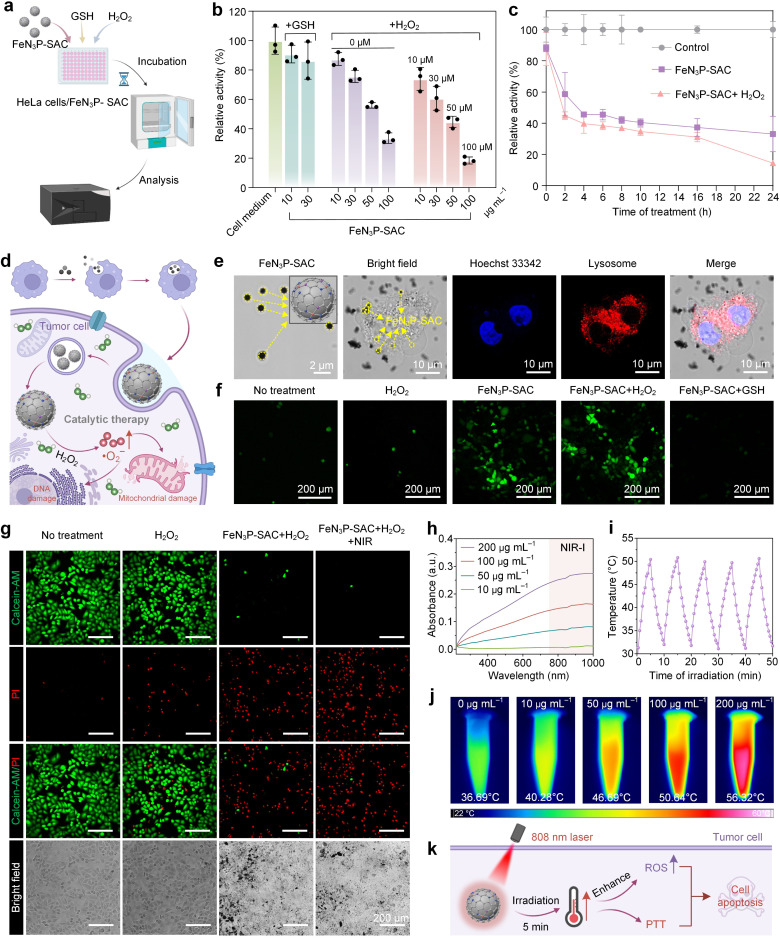
FeN_3_P-SAC inhibits tumor cell growth *in vitro*. (a) Schematic of cell viability investigation with FeN_3_P-SAC, GSH, and H_2_O_2_. (b) Cell viability of HeLa cervical cells after incubation with FeN_3_P-SAC, FeN_3_P-SAC + GSH, and FeN_3_P-SAC + H_2_O_2_ for 24 h over a dose range of 0–100 μg mL^−1^. (c) Time-dependent cell death of HeLa cells after incubation with the control, FeN_3_P-SAC, and FeN_3_P-SAC + H_2_O_2_. (d) Schematic diagram of tumor catalytic therapy of FeN_3_P-SAC. (e) Bright-field images of FeN_3_P-SAC as well as CLSM images of the intracellular uptake of FeN_3_P-SAC in lysosomes. The black spot is FeN_3_P-SAC and yellow dashed circles indicate the distribution of FeN_3_P-SAC within the HeLa cells. Scale bars, 10 μm. (f) CLSM images of reactive oxygen species in the HeLa cells after different treatments. HeLa cells were stained with H_2_DCFDA and the change in fluorescence was observed by CLSM. Scale bars, 200 μm. (g) CLSM images of calcein-AM (green, live cells) and PI (red, dead cells) co-stained HeLa cells treated with PBS, H_2_O_2_, FeN_3_P-SAC + H_2_O_2_, and SACs + NIR (808 nm NIR laser irradiation), respectively. Scale bars, 200 μm. (h) UV-Vis-NIR spectra of FeN_3_P-SAC at different concentrations. (i) Photothermic stability of FeN_3_P-SAC (100 μg mL^−1^) upon 808 nm laser irradiation (5 min, 1.5 W cm^−2^) for 5 on/off cycles. (j) The photothermal effect of FeN_3_P-SAC at different concentrations after being irradiated with an 808 nm laser (1.5 W cm^−2^) for 5 min. (k) Schematic of FeN_3_P-SAC-mediated enhanced photothermal therapy. For (b) and (c), *n* = 3 independent measurements, with data presented as means ± s.d.

The excellent POD-like catalytic activity of FeN_3_P-SAC under acidic conditions indicated that FeN_3_P-SAC could serve as an effective nanocatalyst for tumor catalytic therapy. Therefore, the ability of FeN_3_P-SAC to inhibit tumor cell growth *in vitro* was evaluated using a standard cell-counting kit-8 (CCK-8) cytotoxicity assay (Fig. 5a). FeN_3_P-SAC demonstrated concentration-dependent and time-dependent HeLa cervical cell death (Fig. 5b and c). With the increase of FeN_3_P-SAC concentration from 0 μg mL^−1^ to 100 μg mL^−1^, the HeLa cell viability significantly decreased. Notably, at an FeN_3_P-SAC concentration of 100 μg mL^−1^, the HeLa cell viability was about 33.65%, indicating the effective suppression of HeLa cells *in vitro* by FeN_3_P-SAC. In addition, no significant difference was observed with or without H_2_O_2_ addition (10–100 μM). Conversely, the introduction of antioxidants such as glutathione (GSH) into HeLa cell cultures with FeN_3_P-SAC resulted in negligible cytotoxicity, indicating the biocompatibility of FeN_3_P-SAC (Fig. 5b).

Subsequently, we postulated the mechanisms for tumor catalytic therapy of FeN_3_P-SAC, suggesting that these SACs were phagocytosed by tumor cells to induce oxidative cell death by catalytically generating large amounts of ROS (·O_2_^−^) to consume GSH under the acidic lysosomal environment (Fig. 5d). As predicted, CLSM images showed that treatment of HeLa cells with FeN_3_P-SAC resulted in successful internalization of the SACs, where FeN_3_P-SAC localized in the lysosomes led to cell swelling and induced apoptosis (Fig. 5e). To compare the content of cytosolic ROS in HeLa cells after phagocytosis of FeN_3_P-SAC, we employed CLSM by utilizing the fluorescent probe H_2_DCFDA.^46^ Notably, there was no substantial difference in ROS accumulation in FeN_3_P-SAC-treated cells with or without the addition of exogenous H_2_O_2_ (Fig. 5f), indicating that endogenous H_2_O_2_ from cellular metabolism is sufficient to activate FeN_3_P-SAC-mediated intracellular catalytic reactions. Additionally, GSH addition significantly reduced the ROS, consistent with the *in vitro* CCK-8 cytotoxicity assay results.

Besides, the inhibitory effects of FeN_3_P-SAC on HeLa cells were further evaluated using live/dead cell staining.^47^ The vast majority of regions in the no treatment and H_2_O_2_ groups showed green fluorescence (living cells), while red fluorescence (dead cells) appeared in the FeN_3_P-SAC with H_2_O_2_ experimental group (Fig. 5g). Based on these results, the FeN_3_P-SAC-mediated intracellular catalytic reaction alone did not result in complete HeLa cell death. To enhance the inhibitory effect, we tried to apply laser irradiation as an additional treatment, which resulted in strong red fluorescence in all treated regions (Fig. 5g, right). This result provided preliminary evidence that FeN_3_P-SAC possesses a certain degree of photothermal properties in addition to catalytic activity.

In order to further clarify the photothermal contribution of FeN_3_P-SAC, we conducted additional characterization of SACs. The water solution of FeN_3_P-SAC exhibits broad absorption ranging from the UV to the NIR region (Fig. 5h). Under 808 nm laser irradiation, FeN_3_P-SAC exhibited an obvious time- and concentration-dependent temperature increase compared to the control (Fig. S22†). For example, the temperature can be elevated by about 23.5 °C under 808 nm laser irradiation at a power density of 1.50 W cm^−2^ for 5 min with FeN_3_P-SAC at 100 μg mL^−1^. Additionally, the calculated average photothermal conversion efficiency (*η*) of FeN_3_P-SAC at 808 nm was determined to be 67.3%, which underscored the remarkable ability of FeN_3_P-SAC to efficiently convert laser energy into heat, making it a potent candidate for photothermal therapy (PTT) (Fig. S23†).^47^ FeN_3_P-SAC was also stable enough to maintain the same photothermal effect even after repeated irradiation for 5 cycles (Fig. 5i and S24†). The excellent photothermal performance of FeN_3_P-SAC was further evidenced by infrared thermal images, with the concentration-dependent heating demonstrating the effective conversion of light energy into thermal energy (Fig. 5j).

To investigate the influence of the photothermal effect on enhancing nanocatalytic activities, we proceeded to examine the catalytic performance of FeN_3_P-SAC following treatment with 808 nm laser irradiation. The POD-like activity of FeN_3_P-SAC showed an increasing trend after laser irradiation (Fig. S25†). This result proved that FeN_3_P-SAC has considerable photothermal performance and can effectively enhance catalytic activities. To sum up, FeN_3_P-SAC serves as a promising nanocatalyst for cascade catalysis and photothermal therapy to induce apoptosis in tumor cells (Fig. 5k).

## Conclusions

In summary, we have developed a facile and efficient PIA strategy for achieving the surface immobilization of single atoms on heteroatom-doped carbon nanospheres, which can be used for nanocatalytic therapy. Single atoms on the surface were prepared by a carbonization process of an MPN-coated PCPP template, which converts the core distributed PCPP templates to N/P-doped carbon and subsequently induces the interfacial chemical coordination between N/P and Fe atoms confined at the interface between N/P-doped carbon and MPNs, forming FeN_3_P-SAC on the surface of N/P-doped carbon nanospheres. The as-obtained FeN_3_P-SAC can possess the fundamental properties of natural enzymes to achieve the significant enhancement of POD-like activity, which can convert a high content of H_2_O_2_ to toxic ROS such as ·O_2_^−^. In addition, FeN_3_P-SAC will further enhance catalytic activities under the excellent photothermal effect of exogenous NIR irradiation. As a proof of concept, the biocatalytic effect on the combined catalytic and photothermal therapeutic modality of FeN_3_P-SAC has been demonstrated *in vitro*. This work not only provides valuable insights into the surface-immobilized design of SACs based on the PIA strategy but also advances our understanding of the coordination environment effect from the support, which will broaden the potential application of bioinspired SACs in biocatalysis.

## Data availability

Data supporting this article have been included as part of the ESI.†

## Author contributions

J. G., Y. Z., and Y. H. conceived the project. Y. Z. designed, conducted, and analyzed the majority of experiments. J. G. and Y. H. supervised the project. Y. J. assisted in peroxidase-like activity and kinetics tests. G. Y. and Y. W. helped with the TEM characterization and provided the necessary resources. Z. W. constructed the structural models. Y. P. assisted in the XRD characterization. S. L. provided suggestions for figure design. W. L. provided the necessary resources. Y. Z. and Y. H. drafted the manuscript. All the authors discussed the results and commented on the manuscript.

## Conflicts of interest

There are no conflicts to declare.

## Supplementary Material

SC-OLF-D4SC07775J-s001
