# Supplementary material for: Surface immobilization of single atoms on heteroatom-doped carbon nanospheres through phenolic-mediated interfacial anchoring for highly efficient biocatalysis
Source: Chem Sci. 2025 Jan 20;16(8):3479–89. doi: 10.1039/d4sc07775j (PMC11770272; doi:10.1039/d4sc07775j)
Supplement: SC-016-D4SC07775J-s001 [file SC-016-D4SC07775J-s001.pdf]

Supplementary Information (SI) for Chemical Science.
This journal is © The Royal Society of Chemistry 2025

Supplementary Information

Surface immobilization of single atoms on heteroatom-doped carbon nanospheres through phenolic-mediated interfacial anchoring for highly efficient biocatalysis

Yajing Zhang, Yunxiang He, Yun Jiao, Guobin Yang, Yiran Pu, Zhangmin Wan, Shuyun Li, Yanchao Wu, Wen Liao and Junling Guo*

*Corresponding authors: E-mail: junling.guo@scu.edu.cn, junling.guo@ubc.ca (J.G.).

Section S1. Experimental Procedures

S1.1 General Materials

Tannic acid (TA) and tris(hydroxymethyl)aminomethane (Tris) were purchased from Sigma-Aldrich (USA). Horseradish peroxidase (HRP) and iron(II,III)oxide (Fe_3O_4) were purchased from Aladdin (China). Iron(III) chloride hexahydrate ($FeCl_3 \cdot 6H_2O$), 3,3’,5,5’-tetramethylbenzidine (TMB), 5,5-Dimethyl-1-pyrrolineN-oxide (DMPO), phloroglucinol, phosphonitrilic chloride trimer (HCCP), dimethyl sulfoxide (DMSO), iron(II) phthalocyanine (FePC), and Fe powder were purchased from Adamas life (China). Iron (III) oxide (Fe_2O_3) and triethylamine (TEA) were purchased from Macklin Biochemical Technology Co., Ltd (Shanghai, China). Iron phosphide (Fe_2P) was purchased from Rhawn Chemical Reagent Co., Ltd. (Shanghai, China). Acetonitrile and hydrochloric acid (HCl) were purchased from Chron Chemical Co., Ltd. (China). Ethanol, acetic acid, sodium acetate anhydrous, and sodium hydroxide were purchased from Jinshan Chemical Reagent Co., Ltd (Chengdu, China). Cell counting kit-8 (CCK-8), Calcein/PI Live/Dead assay kit, Hoechst 33342, Lyso-Tracker Red, cyanine7-human serum albumin (Cy7-HSA), and reactive oxygen species assay kit (2′,7′-dichloroflfluorescin diacetate, H_2DCFDA) was purchased from Beyotime (China). Phosphate buffered saline (PBS) was purchased from Gibco (USA). All of these materials were used as received. High-purity Milli-Q (MQ) water with a resistivity of 18.2 MΩ cm was obtained from an inline ill pore Rio’s/Origin water purification system. All the chemicals were A.R. grade. All chemicals were used as received without further purification.

S1.2 General Characterization

Instruments

Scanning electron microscopy (SEM) of PCPP, PCPP/Fe-TA, and FeN_3P-SAC was performed on a JEOL JSM-7500F SEM instrument. Transmission electron microscopy (TEM), high-resolution TEM (HRTEM), selected area electron diffraction (SAED) pattern, and energy dispersive X-ray spectroscopy (EDX) were performed on a Tecnai G2 F20 S-TWIN instrument, operating at a voltage of 200 kV (FEI USA, Inc.). The atomic structure of FeN_3P-SAC was characterized using a Spectra300 TEM operated at 200 keV and equipped with double spherical aberration (Cs) correctors. Zeta-potentials and size distribution of the PCPP and PCPP/Fe-TA were obtained by dynamic light scattering (DLS) on Zetasizer Nano ZSP (Malvern, UK). Fourier transform-infrared spectra (FT-IR) of monomers, PCPP, and PCPP/Fe-TA were recorded on a Nicolet iS10 instrument in the range of 500–4000 cm^{-1} using the KBr pellet technique. UV-Visible absorption measurements of monomers, PCPP, and PCPP/Fe-TA were conducted on a Lambda 1050 spectrophotometer. X-ray photoelectron spectroscopy (XPS) results of PCPP/Fe-TA and FeN_3P-SAC were conducted on an AXIS Ultra DLD instrument. X-ray absorption fine structure (XAFS) spectroscopy of FeN_3P-SAC was carried out using the Rapid XAFS 2M (Anhui Absorption Spectroscopy Analysis Instrument Co., Ltd.) by transmission (or

fluorescence) mode at 20 kV and 20 mA, and the Si (531) spherically bent crystal analyzer with a radius of curvature of 500 mm was used for Fe. The X-ray diffraction (XRD) patterns of corresponding samples were collected using a Rigaku Ultima IV X diffractometer operating at 40 kV and 40 mA with Cu $K\alpha$ radiation (λ = 1.5406 Å). The Raman spectra of PCPP-800 and FeN_3P-SAC were collected on a (Renishaw) Raman spectrometer using a 532 nm laser. The specific surface area of corresponding samples was analyzed using nitrogen adsorption-desorption isotherms and the Brunauer-Emmett-Teller (BET) method (Micromeritics ASAP 2460 Version 3.01). The thermal gravimetric analysis (TGA) of corresponding samples was conducted on the TG209F3 instrument, which was annealed in N_2 at 20 °C min^{-1} up to 1000 °C. The exact Fe contents of FeN_3P-SAC were determined by inductively coupled plasma optical emission spectrometry (ICP-OES) measurement.

Catalytic performance evaluation and CCK-8 assay of FeN_3P-SAC were performed with an Infinite Nano microplate reader (Tecan Group, Switzerland). Reactive oxygen species (ROS) catalyzed by FeN_3P-SAC were detected by Bruker EMX plus X-band EPR (Bruker Ltd, USA). Fluoresce images of PCPP, Cy7-HSA@PCPP/Fe-TA, as well as HeLa cells treated with FeN_3P-SAC were obtained by confocal laser scanning microscopy (CLSM) (Leica, Germany). The photothermal temperature of FeN_3P-SAC suspension was recorded by Fluke Ti480 PRO Infrared Camera.

S1.3 Synthesis of PCPP Templates

PCPP templates were firstly fabricated by a straightforward condensation polymerization method according to the reported procedure. The mechanistic diagram of the synthesis is depicted in **Figure S1**.[1] Briefly, phloroglucinol (281 mg, 2.23 mM) and HCCP (204 mg, 0.59 mM) were dissolved in 150 mL of acetonitrile to obtain a clear solution. Following this, triethylamine (TEA) (8 mL) was rapidly added to the above mixture, resulting in an immediate formation of a milky white solution. The reaction mixture was then subjected to continuous ultrasonication at 40 °C for 5 h. Afterward, these white solids were obtained by centrifugation at 5000 g for 5 min, washed 3 times with ethanol and MQ water, respectively, and dried in a vacuum at 60 °C overnight to yield PCPP powder

S1.4 Preparation of PCPP/Fe-TA

To prepare the PCPP/Fe-TA, we employed a typical MPNs surface coating procedure reported by our group.[2] Initially, 400 mg of PCPP powder was suspended in 100 mL of MQ water and uniformly dispersed via ultrasonication at 160 W and 50 Hz for 30 min. Subsequently, 4 mL of TA (10 mg mL^{-1}) and $FeCl_3 \cdot 6H_2O$ (10 mg mL^{-1}) were added dropwise to the above system, respectively, in which the color of the solution gradually changed from white to purple. Tris buffer solutions (100 mM, pH=8.0) were then added to increase the pH of the suspension, followed by stirring at room temperature for 1 h. The PCPP/Fe-TA core-shell composites were centrifuged (5000 g, 3 min) and washed 3 times with ethanol and MQ water, respectively, until the supernatant became clear

and then dried under vacuum at 60 °C overnight. To avoid particle aggregation, sonication was performed after each wash step. All synthesized products were stored at room temperature (~ 25 °C) for subsequent use.

S1.5 Synthesis of FeN_3P-SAC

The detailed process used to synthesize was described as follows. The as-obtained powder of PCPP/Fe-TA as the catalyst precursor was placed in a quartz boat and then kept at 800 °C for 3 h in a tube furnace with a heating rate of 10 °C min^{-1} under a flowing argon (Ar) atmosphere. When samples cooled to room temperature, the resulting materials were collected and washed with HCl solution (3 M) and MQ water for 3 times and dried at 60 °C in a vacuum overnight. Then, the dried black powder samples were heated to 800 °C and kept under a flowing Ar atmosphere with the heating rate (10 ºC min^{-1}) for 3 h to obtain FeN_3P-SAC.

S1.6 Catalytic Performance Evaluation of FeN_3P-SAC

S1.6.1 Measurement of POD-like activities: The POD-like activities of FeN_3P-SAC were measured by testing the absorbance of oxidized TMB (ox-TMB) at 652 nm by utilization of an Infinite Nano microplate reader. Initially, 75 µL of TMB solution (1.66 mM) was added into 50 µL of FeN_3P-SAC solution (200 µg mL^{-1}) containing 75 µL of H_2O_2 (3.90 mM). TMB solution and TMB+H_2O_2 solution were used as controls. After 5 min, the catalytic oxidation of TMB was studied by the Infinite Nano microplate reader in wavelength-scan mode. Similarly, PCPP-800 was used as a control sample in this experiment. Furthermore, to verify the effect of pH and temperature on POD-like activities of FeN_3P-SAC, we controlled the pH value of the HAc-NaAc buffer (0.2 M, pH 3.6) by adding NaOH solution (4.0 M) or the temperature of by performing the reaction in a water bath to obtain the corresponding POD activity-pH curve and POD activity-temperature curve, respectively. To confirm the concentration-dependent absorbance upon the addition of FeN_3P-SAC, we added the FeN_3P-SAC with different concentrations (0–100 µg mL^{-1}) to a solution of 0.064 mM TMB and 0.915 mM H_2O_2 in HAc-NaAc buffer (0.2 M, pH = 3.6), and we recorded the absorbance at 652 nm in time-scan mode.

S1.6.2 Characterization of the kinetics: We performed kinetic analysis of the FeN_3P-SAC, Fe_3O_4 nanoparticles, and HRP with H_2O_2 and TMB as the substrate by changing the concentration of H_2O_2 at a fixed concentration of TMB (0.62 mM) or changing the concentration of TMB at a fixed concentration of H_2O_2 (1.47 mM) in the presence of FeN_3P-SAC (10 µg mL^{-1}). Unless stated otherwise, all experiments were carried out at room temperature and pH 3.6. The absorbance at 652 nm against the reaction time was plotted to obtain the reaction-time curve. We determined the initial rate (k_1) of change of absorbance at 652 nm by measuring the slope of the initial linear portion of the reaction-time curve.

The initial reaction rates (V_0) of various H_2O_2 or TMB concentrations were calculated by using the initial absorbance-time rate (k_1) and Beer-Lambert Law (1). The Michaelis-Menten curve was produced by plotting the calculated V_0 against

corresponding H_2O_2 or TMB concentrations. The values of K_m and V_m were obtained through the linear double-reciprocal plot (Lineweaver-Burk plot) (3). Furthermore, the value of K_{cat} was calculated by dividing V_{max} by the molar concentration of the FeN_3P-SAC or HRP (4).[3]

$$A = \varepsilon bc \tag{1}$$

$$V_0 = \frac{V_{max} \cdot [S]}{K_m + [S]} \tag{2}$$

$$\frac{1}{V_0} = \frac{k_m}{V_{max}} \cdot \frac{1}{[S]} + \frac{1}{V_{max}} \tag{3}$$

$$K_{cat} = \frac{V_{max}}{[E]} \tag{4}$$

Where A is the absorbance at 652 nm, b is the optical path length of 1 cm, ε and c are the molar absorptivity of the TMB oxidation product (39,000 $M^{-1}cm^{-1}$) and the concentration, respectively. *[S]* denotes the substrate concentration, with the unit mol L^{-1}. *[E]* represents the number of enzyme or iron active sites participating in the reaction, with the unit mol.

S1.6.3 Measurement of durable catalytic performance: 30 μL of FeN_3P-SAC solution (200 μg mL^{-1}), 20 μL of H_2O_2 solution (3.9 mM), and 20 μL of TMB solution (1.66 mM) were sequentially added to a 96-well plate for absorbance measurement at 652 nm in time-scan mode. After 5 min of reaction, an additional 20 μL of H_2O_2 solution and 20 μL of TMB solution were added to the above reaction system, and the absorbance was continued to be measured at 652 nm in time-scan mode. After 5 min of reaction, a similar experimental procedure was continued to demonstrate durable catalytic performance. of FeN_3P-SAC.

S1.6.4 Measurement of catalytic recyclability: 300 μL of FeN_3P-SAC solution (200 μg mL^{-1}), 450 μL of H_2O_2 solution (3.9 mM), and 450 μL of TMB solution (1.66 mM) were sequentially added to a 1.5 mL centrifuge tube. Following centrifugation for 5 mins, 200 μL of the supernatant was transferred to a 96-well plate for absorbance measurement at 652 nm. Meanwhile, the FeN_3P-SAC precipitate was retained for subsequent cycles to assess relative POD-like activity across multiple experimental operations.

S1.6.5 Durability assessment of FeN_3P-SAC: We first detected the leaching of Fe from FeN_3P-SAC in HAc-NaAc buffer solutions (acid, pH 3.6) by using ICP-OES. Specifically, 1.5 mL of FeN_3P-SAC solution (200 μg mL^{-1}) and 4.5 mL of HAc-NaAc buffer solution were sequentially added to a 15 mL centrifuge tube. After treatment for different times, such as 0, 0.5, 1, 5, 10, and 24 h, corresponding centrifuge tubes were

centrifuged to aspirate the supernatant and recover the catalyst. Finally, these supernatants were measured to verify Fe leaching from FeN_3P-SAC by ICP-OES. All experiments were performed three times independently. Subsequently, to further verify less leaching of Fe affects the catalytic activity, we used the untreated catalysts and treated catalysts with Fe leaching to determine the POD-like activity. Briefly, 60 μL of untreated catalysts solution or treated catalysts solution (200 μg mL^{-1}), 90 μL of H_2O_2 solution (3.9 mM), and 90 μL of TMB solution (1.66 mM) were sequentially added to a 96-well plate for absorbance measurement at 652 nm. The control indicates no addition of any samples. All experiments were performed three times independently.

S1.6.6 Electron paramagnetic resonance (ESR) detects the reactive oxygen species (ROS): The production of the superoxide radical ($•O_2^-$) and hydroxyl radical (•OH) catalyzed by FeN_3P-SAC was detected by utilizing 5,5-dimethyl-1-pyrroline N-oxide (DMPO) as a trapping agent. For the determination of •OH, 100 μg FeN_3P-SAC was added into a 1.0 mL HAc-NaAc buffer solution (0.2 M, pH=3.6) containing H_2O_2 (100 mM) and DMPO (50 mM). After vortexed for 5 min, the mixture was transferred to a quartz tube for ESR assay. In contrast, the generation of $•O_2^-$ was also evaluated by an ESR spectrometer using DMPO as a spin-trapping adduct and DMSO as a buffer.

S1.7 Cell Experiments

S1.7.1 Cell culture: HeLa cells were cultured with Dulbecco’s Modified Eagle Medium (DMEM, Sigma) containing 10% fetal bovine serum (FBS, Gibco) and 1% penicillin-streptomycin at 37 °C under an atmosphere of 5% CO_2.

S1.7.2 Cell viability assay: We detected the cytotoxicity of FeN_3P-SAC by using a CCK-8 cell viability kit assay. Briefly, the HeLa cells (1×10^4 cells per well) were plated in 96-well plates and cultured for 24 h for adherence before the addition of FeN_3P-SAC with different concentrations (with or without H_2O_2 and GSH). On each plate, blank wells with media are background, and the wells with DMEM-treated cells only were defined as 100% viability. FeN_3P-SAC, H_2O_2, and GSH were diluted in DMEM medium and then added into the cells. After incubation for different periods, the medium was replaced with 100 μL fresh DMEM medium, and 10 μL CCK-8 solution was added. Finally, according to the manufacturer’s instructions, we measured the absorbance at 450 nm by a microplate reader. All experiments were independently performed three times.

S1.7.3 Live-dead cell staining assay: HeLa cells were seeded in confocal laser dishes at a density of 1×10^4 per dish and further cultured for 12 h. Then, the medium was replaced with fresh DMEM medium containing various samples (100 μM H_2O_2, 100 μg mL^{-1} FeN_3P-SAC+100 μM H_2O_2, 100 μg mL^{-1} FeN_3P-SAC+100 μM H_2O_2+NIR). These confocal laser dishes were incubated for another 24 h at 37 °C. For 100 μg mL^{-1} FeN_3P-SAC+100 μM H_2O_2+NIR group, cells were exposed to an 808 laser (1.50 W cm^{-2}) for 5 min. Among them, cells treated with PBS serve as the control. After that,

cells were dyed with Calcein-AM/PI for 30 min. Finally, the cells were washed 3 times with PBS and observed by CLSM.

S1.7.4 Uptake of FeN_3P-SAC by Cells: The cellular uptake and distribution of FeN_3P-SAC were evaluated in HeLa cells by CLSM. HeLa cells were seeded in confocal laser dishes (1×10^4 per dish) and incubated overnight. After replacing the medium with 1 mL fresh DMEM medium containing 100 μg mL^{-1} FeN_3P-SAC, cells were further incubated for 24 h. After 24 h, the medium was removed, and the cells were stained with fresh DMEM medium containing Hoechst 33342 (blue, nucleus) and Lyso-Tracker Red (red, lysosome) for 30 min. Meanwhile, FeN_3P-SAC was added to the PBS solution as a control. Finally, these samples were imaged with CLSM.

S1.7.5 Cellular ROS assay: For the intracellular generation of ROS, we used the H_2DCFDA (a fluorescent probe) to conduct this test. Specifically, HeLa cells were seeded in confocal laser dishes (1×10^4 per dish) and incubated overnight. After adhesion, the medium was replaced with fresh DMEM medium containing various samples (100 μM H_2O_2, 100 μg mL^{-1} FeN_3P-SAC, 100 μg mL^{-1} FeN_3P-SAC+100 μM H_2O_2, and 100 μg mL^{-1} FeN_3P-SAC+100 μM GSH), then further incubated for another 12 h at 37°C. After washing with PBS, we stained the HeLa cells with the fluorescence probe 10 μM H_2DCFDA in serum-free medium for 30 min at 37 °C. Finally, these cells were washed with PBS and observed for fluorescence by CLSM.

S1.8 Investigation of Photothermal Performance

***S1.8.1 Photothermal performance of FeN_3P-SAC*:** 1.5 mL FeN_3P-SAC suspension with different concentrations (10, 50, 100, and 200 μg mL^{-1}) and pure water as control were placed into the irradiation of an 808 nm laser (1.50 W cm^{-2}) for 6 min, respectively. Meanwhile, the laser-caused temperature increases and images were monitored by a Fluke Ti480 Pro thermal imaging camera. In addition, the photothermic stability of FeN_3P-SAC (100 μg mL^{-1}) upon 808 nm laser irradiation (5 min or 10 min, 1.5 W cm^{-2}) was studied by 5 consecutive on/off photothermal cycles.

S1.8.2 Calculation of the photothermal conversion efficiency: To confirm the photothermal conversion effect, the FeN_3P-SAC suspension (100 μg mL^{-1}, 1.5 mL) was irradiated using an 808 nm laser (1.50 W cm^{-2}) over a period of 300 s, followed by shutting off the NIR laser and cooling process in nature. In this process, a Fluke Ti400 thermal imaging camera was exploited to monitor the temperature during laser irradiation and cooling.

We calculated the photothermal conversion efficiency (η) of FeN_3P-SAC using the following formula:[4]

$$\eta = \frac{hs\Delta T_{max} - Q_{Dis}}{I(1-10^{-A_{808}})} \qquad (1)$$

Where h means the heat transfer coefficient, s refers to the surface area of the container. ΔT_{max} is the maximum temperature change. Q_{Dis} represents heat dissipated from the laser mediated by the solvent and container. I stand for laser power density and A_{808} is the absorbance at 808 nm. hs is determined based on Eq (2) as follows:

$$hs = \frac{mC_{water}}{\tau_s} \qquad (2)$$

Where m is the mass of the solution containing the photoactive material, C_{water} is the heat capacity (4.2 J g^{-1}) of pure water, and τ_s is the associated time constant. To determine τ_s, the following Eq (3) is required:

$$t = -\tau_s(\ln\theta) \qquad (3)$$

Where θ is the ratio of ΔT to ΔT_{max}. From the inset of **Figure S23**, τ_s is calculated to be 210.7 s.

Section S2. Supplementary Information Figures

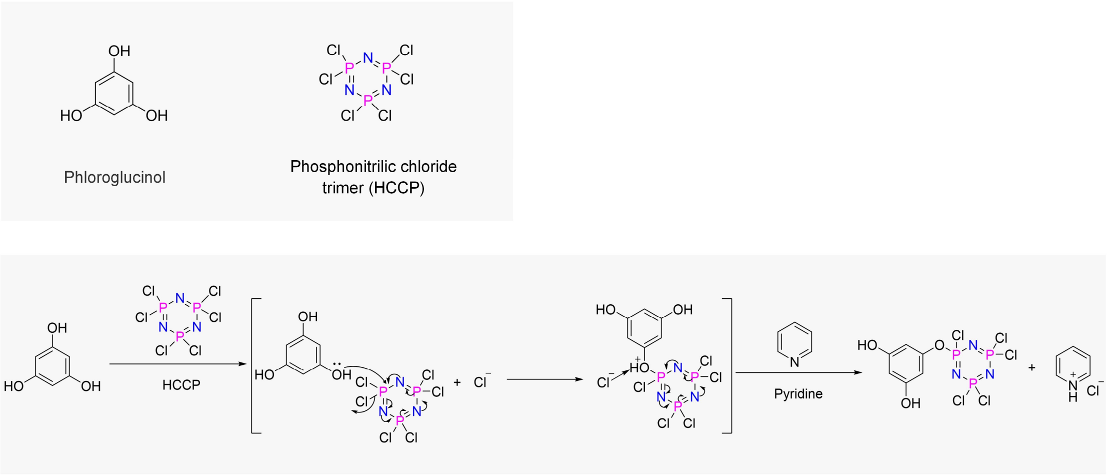

Figure S1. (a) Chemical structures of phloroglucinol and HCCP. (b) The mechanism of PCPP template synthesis by the polycondensation reaction.

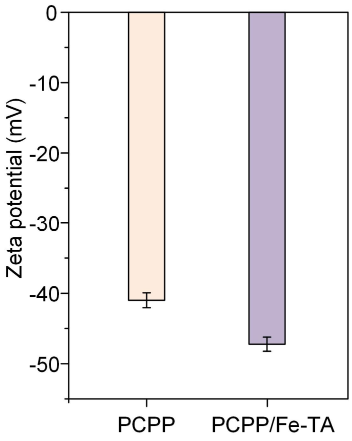

Figure S2. Zeta potential of PCPP and PCPP/Fe-TA in water.

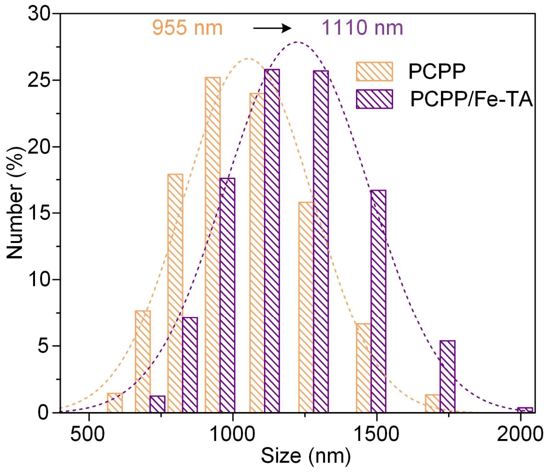

Figure S3. Hydrodynamic particle size distribution of PCPP and PCPP/Fe-TA in water.

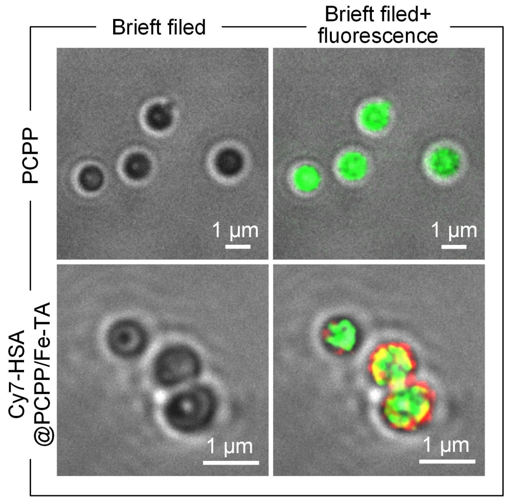

Figure S4. CLSM images of PCPP and Cy7-HSA@PCPP/Fe-TA. Scale bars, 1 μm.

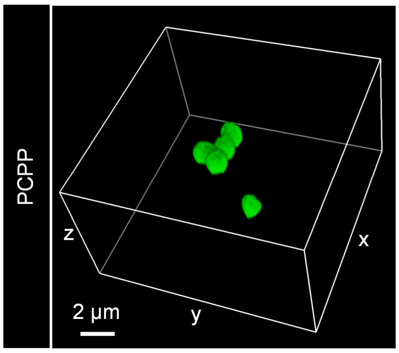

Figure S5. 3D CLSM image of PCPP. Scale bar, 2 μm.

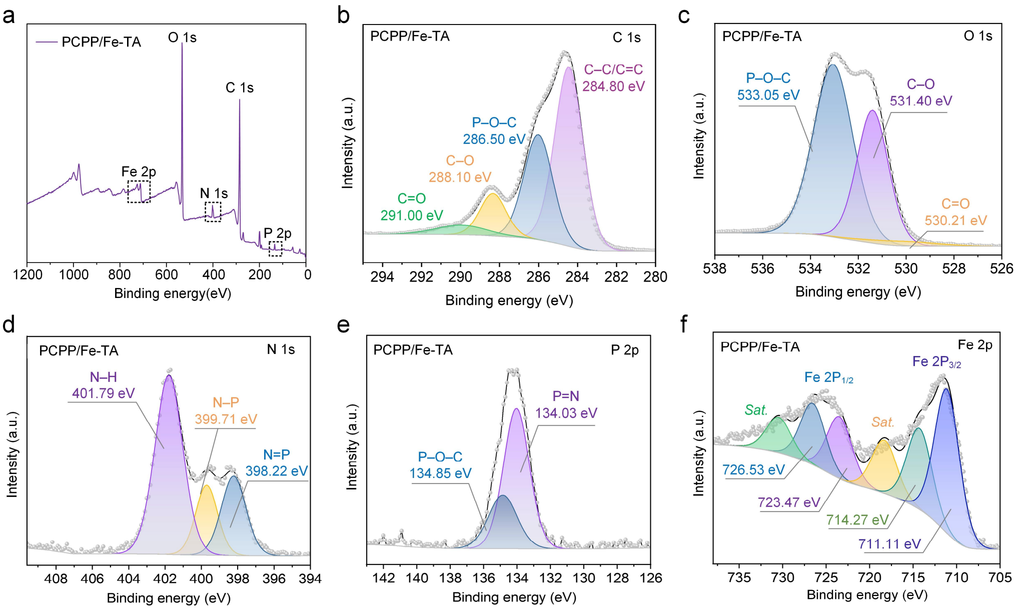

Figure S6. (a) XPS survey spectra of PCPP/Fe-TA. (b–f) High-resolution XPS spectra of N 1*s* (b), O 1*s* (c), N 1*s* (d), P 2*p* (e), and Fe 2*p* (f) for PCPP/Fe-TA. Specifically, Figure. S6b showed the high-resolution C 1*s* peaks consisting of four bands with peak positions, in which the two dominant peaks with binding energy data of 286.50 eV and 291.00 eV represent the P–O–C bonds from the PCPP template and C=O bonds from the Fe-TA nanocoating, respectively. In addition, P–O–C bonds and C=O bonds of high-resolution O 1*s* and P 2*p* peaks also further demonstrated the successful polymerization of monomers (HCCP and phloroglucinol) and the nanocoating formation based on the phenolic group coordination (Figure S6c and S6e). Among them, the P–N bonds and P=N bonds in the high-resolution N 1*s* peaks revealed the presence of HCCP (Figure S6d). In particular, the high-resolution Fe 2*p* peaks suggested that iron in the nanocoating was primarily present as Fe^{3+}/Fe^{2+} owing to the presence of the Fe 2*p* peaks at binding energies of 726.53 eV, 711.11 eV, 723.47 eV, and 714.27 eV.

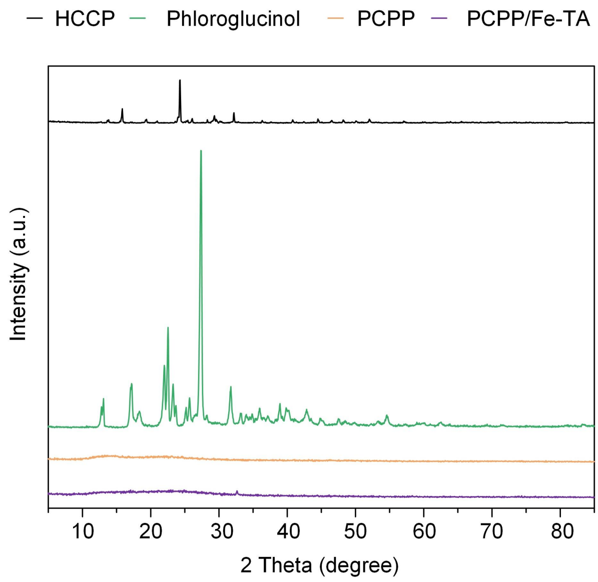

Figure S7. Powder XRD patterns of monomers (HCCP and phloroglucinol), PCPP, and PCPP/Fe-TA.

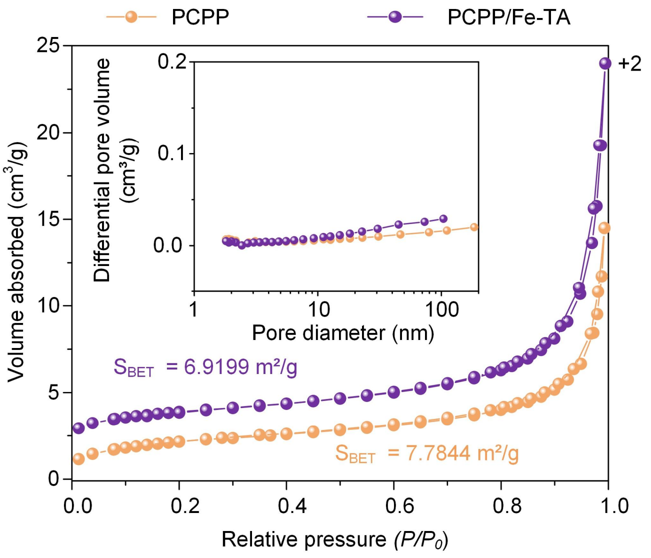

Figure S8. N_2 adsorption-desorption isotherms of PCPP and PCPP/Fe-TA. The inset shows pore size distribution.

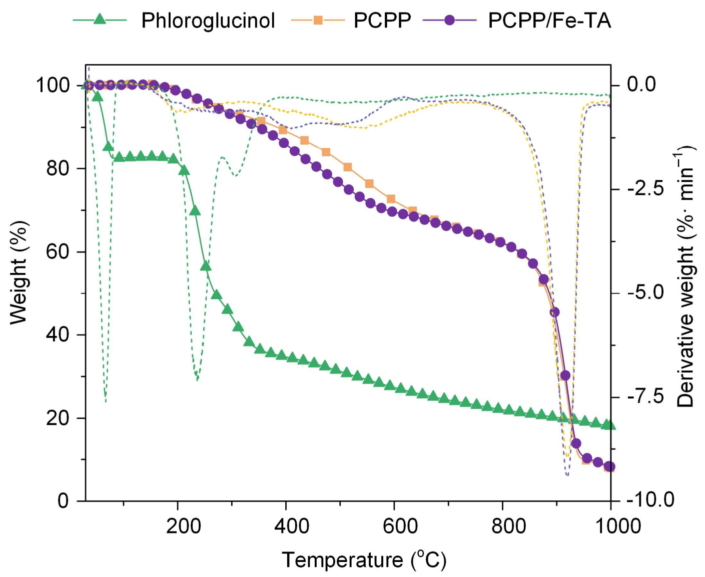

Figure S9. TGA and DTG curves of phloroglucinol, PCPP, and PCPP/Fe-TA under N_2 atmosphere.

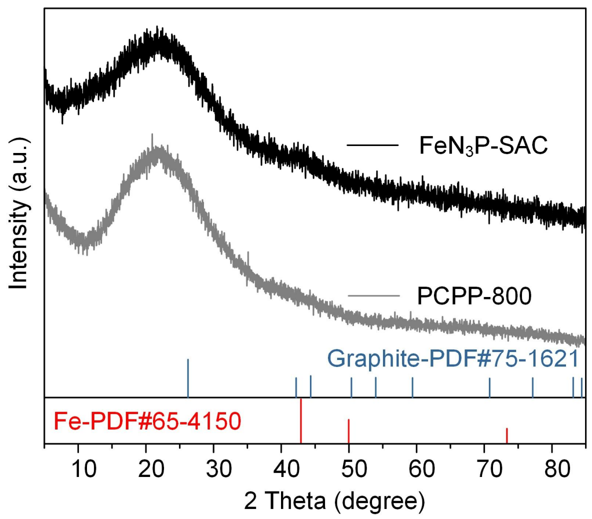

Figure S10. Powder XRD patterns of PCPP-800, FeN_3P-SAC, and standard graphite and Fe.

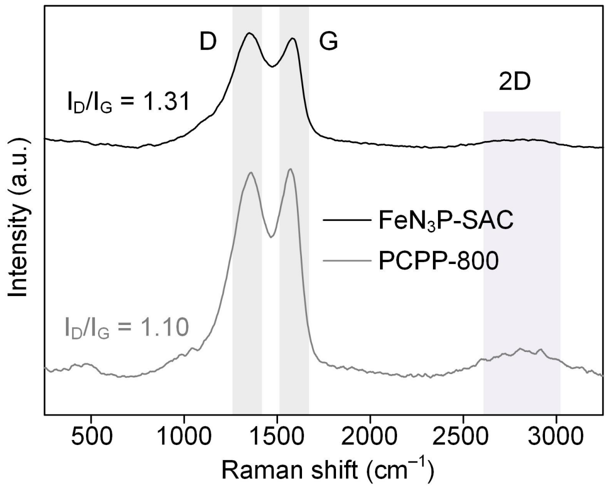

Figure S11. Raman spectra of PCPP-800 and FeN_3P-SAC.

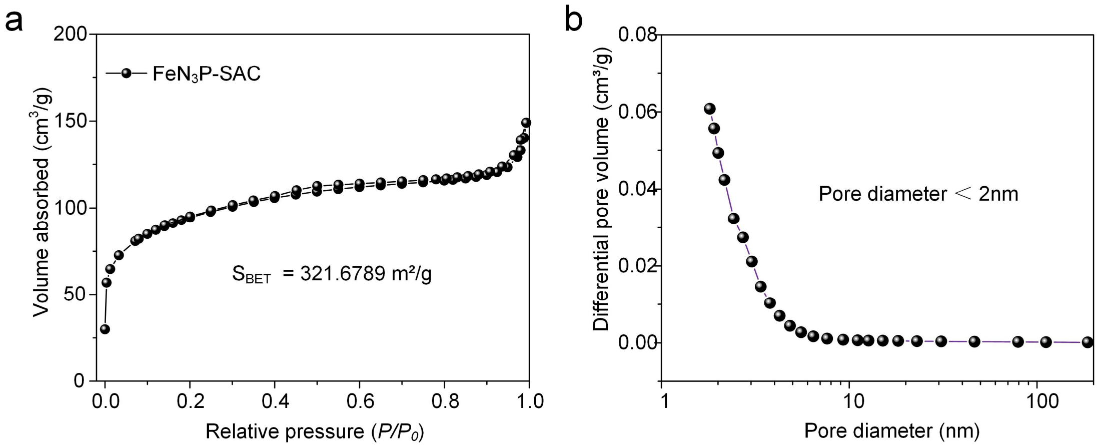

Figure S12. N_2 adsorption-desorption isotherms (a) and pore size distribution (b) of FeN_3P-SAC.

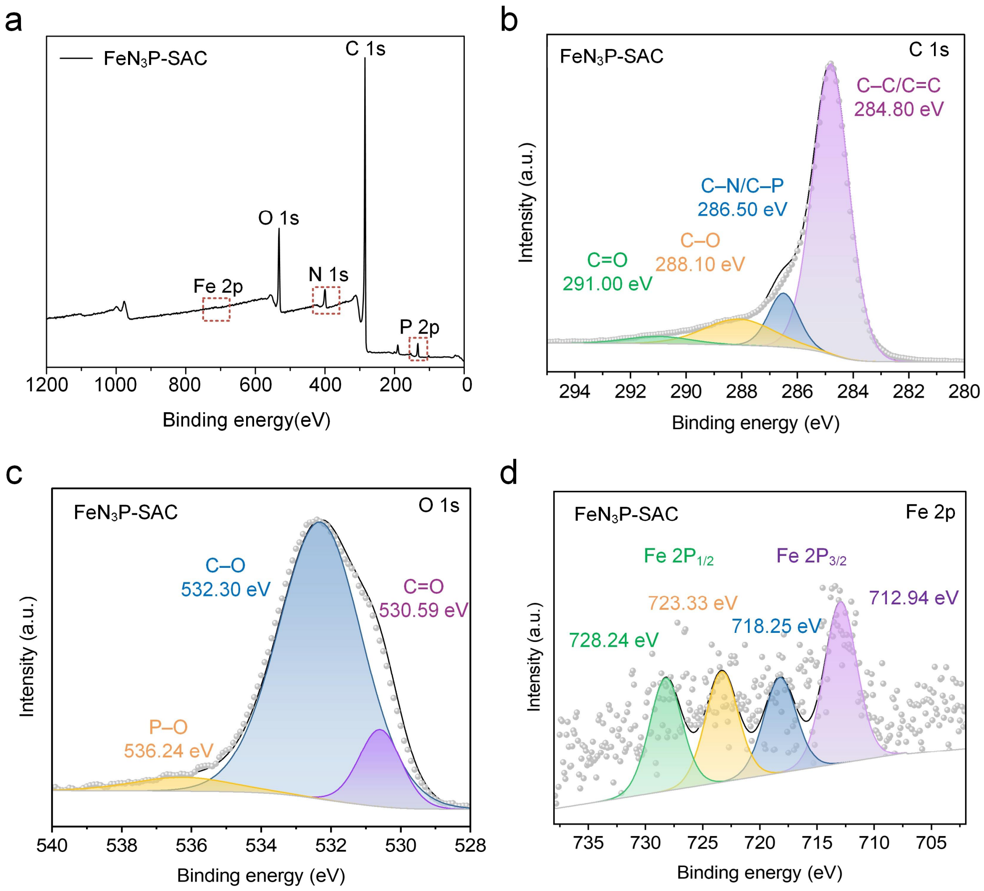

Figure S13. (a) XPS survey spectra of FeN_3P-SAC. (b–d) High-resolution XPS spectra of C 1s (b), O 1s (c), and Fe 2p (f) for FeN_3P-SAC.

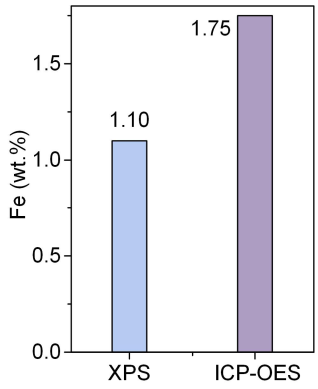

Figure S14. Fe content of FeN_3P-SAC obtained by XPS and ICP-OES.

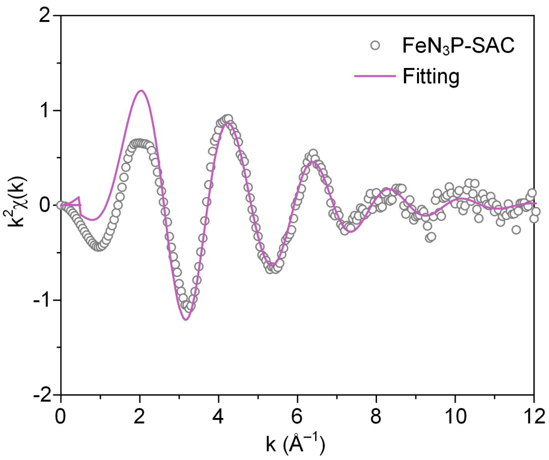

Figure S15. FT-EXAFS fitting curves fitting curve of FeN_3P-SAC in *k* space.

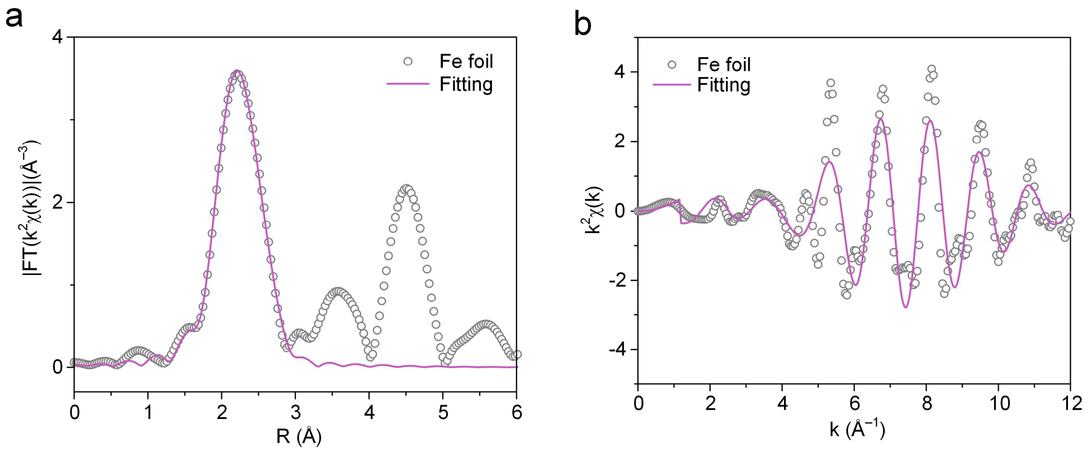

Figure S16. FT-EXAFS fitting curves fitting curve of Fe foil in (a) *R* space and (b) *k* space.

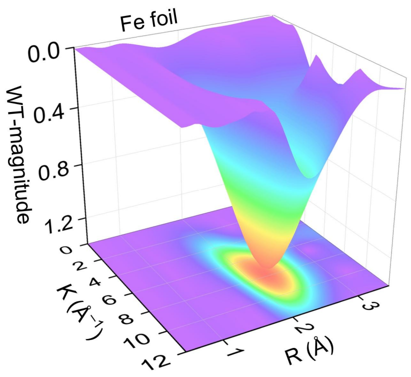

Figure S17. WT EXAFS contour plots of Fe *K*-edge for Fe foil.

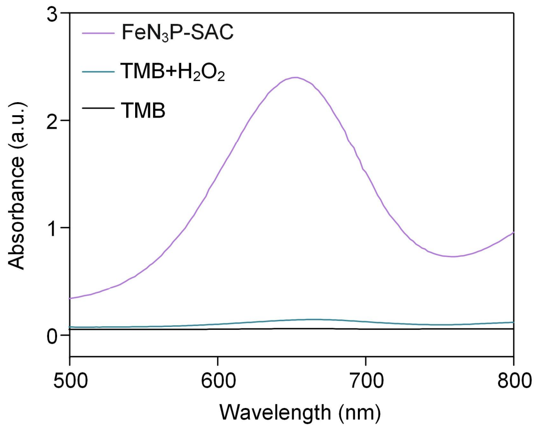

Figure S18. UV-Vis spectra of different solutions for TMB oxidation. The H_2O_2 and TMB concentrations were 1.462 mM and 0.623 mM, respectively. The FeN_3P-SAC concentration was 50 μg mL^{-1}.

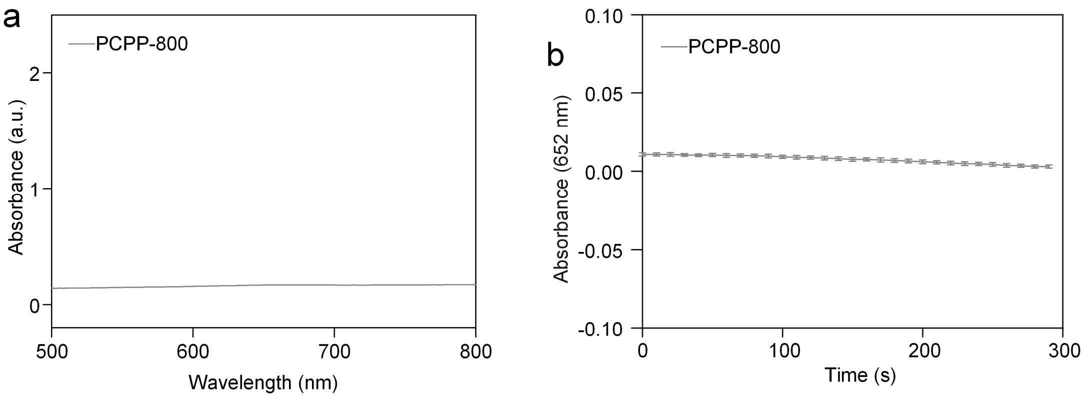

Figure S19. Control experiments show that PCPP-800 demonstrated no catalytic activity toward H_2O_2 and TMB. (a) UV-Vis spectra of PCPP-800 (50 μg mL^{-1}) for TMB oxidation under H_2O_2 (1.462 mM) and TMB (0.623 mM). (b) Reaction-time curves of the TMB colorimetric reaction catalysed by PCPP-800 (10 μg mL^{-1}) under H_2O_2 (1.462 mM) and TMB (0.465 mM) (n = 3 independent measurements, data presented as means ± s.d.).

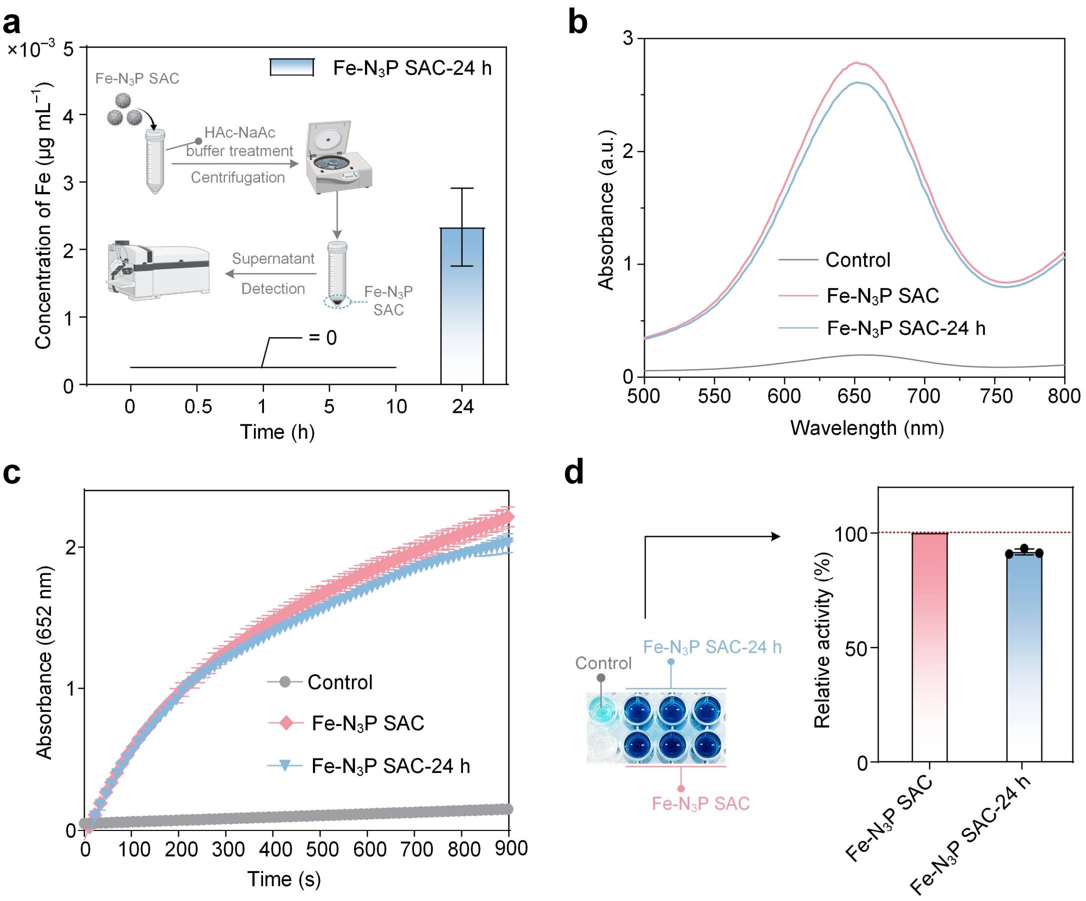

Figure S20. Durability assessment of FeN3P-SAC treated with HAc-NaAc buffer (pH 3.6) for 24 h. (a) Real-time monitoring of Fe leaching from FeN3P-SAC in HAc-NaAc buffer by ICP-OES. FeN3P-SAC-24 h indicates that FeN3P-SAC was treated with HAc-NaAc buffer for 24 h. The inset shows the schematic of Fe leaching detection from the FeN3P-SAC. (b) UV-Vis spectra of control, FeN3P-SAC (50 μg mL^{-1}), and FeN3P-SAC-24 h (50 μg mL^{-1}) for TMB oxidation (900 s) under H_2O_2 (1.462 mM) and TMB (0.623 mM). (c) Reaction-time curves of the TMB colorimetric reaction catalyzed by control, FeN3P-SAC (50 μg mL^{-1}), and FeN3P-SAC-24 h (50 μg mL^{-1}) under H_2O_2 (1.462 mM) and TMB (0.623 mM). (d) Optical photographs and corresponding POD-like activities of different samples reacted for 900 s in (c) reaction conditions. The control indicates no addition of samples. For (a), (c), and (d): n = 3 independent measurements, data presented as means ± s.d.

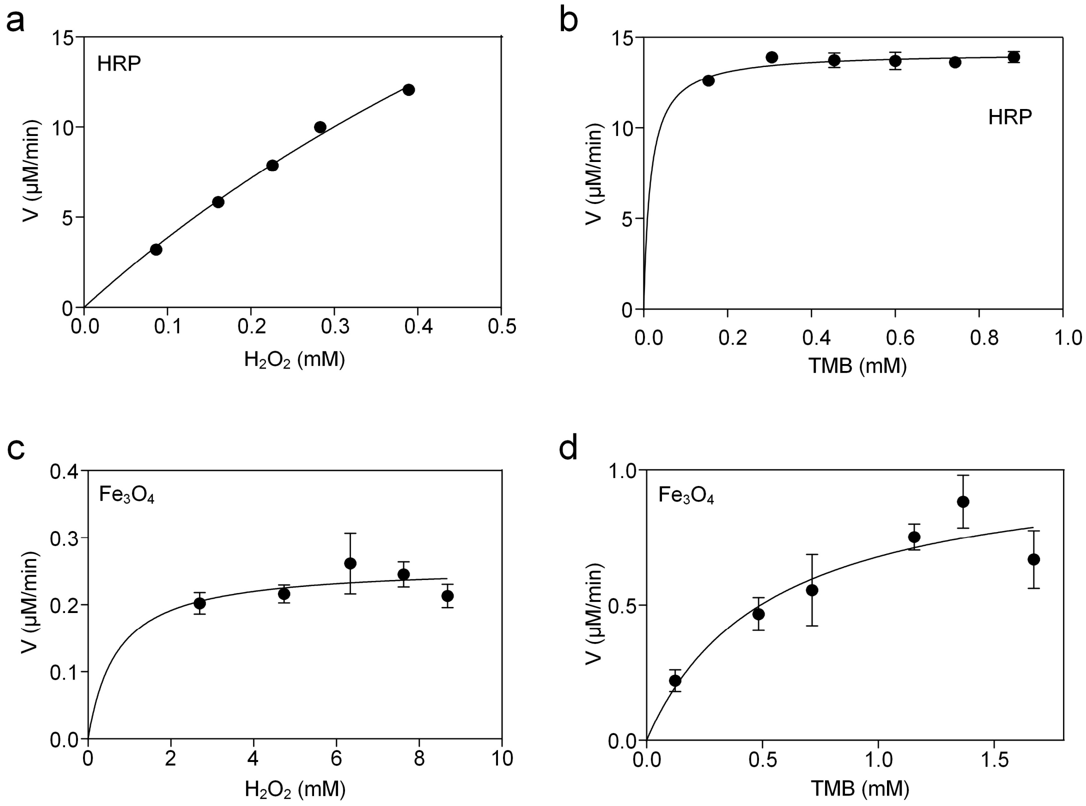

Figure S21. Characterization of the catalytic kinetics of natural HRP and Fe_3O_4. The initial reaction velocity (v) was measured in HAc-NaAc buffer at pH 3.6. (a, c) When the concentration of H_2O_2 was varied, the concentration of TMB used for HRP was 0.154×10^{-3} M, and for Fe_3O_4. was 0.467×10^{-3} M. (b, d) When the concentration TMB was varied, the concentration of H_2O_2 used for HRP was 0.489×10^{-3} M, and for Fe_3O_4. was 0.141 M ($n = 3$ independent measurements, data presented as mean ± s.d.).

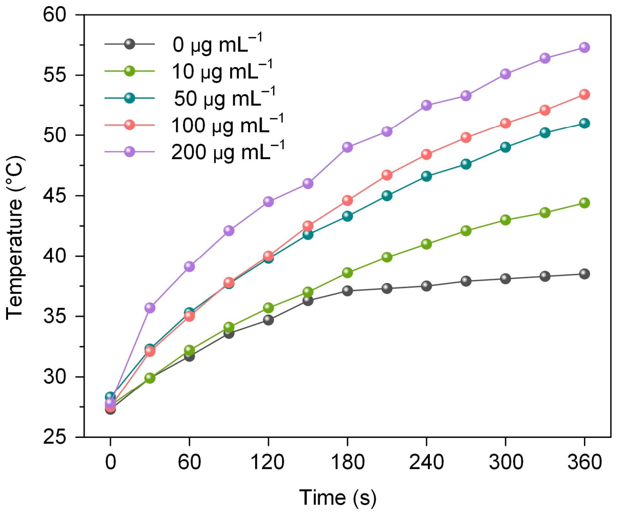

Figure S22. Photothermal heating curves for FeN_3P-SAC dispersions at different dispersion concentrations (808 nm, 1.5 W cm^{-2}).

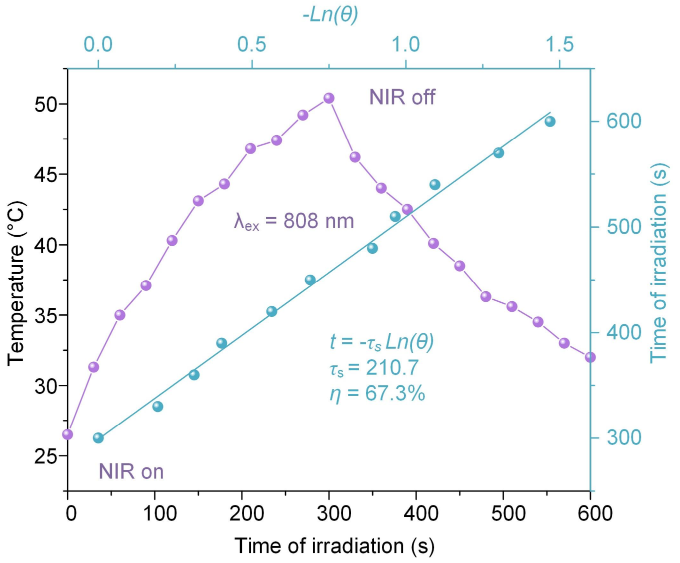

Figure S23. Photothermal performance of FeN_3P-SAC (100 μg mL^{-1}) by cooling to room temperature with linear analysis. Calculation of the photothermal conversion efficiency of FeN_3P-SAC η = 67.3% .

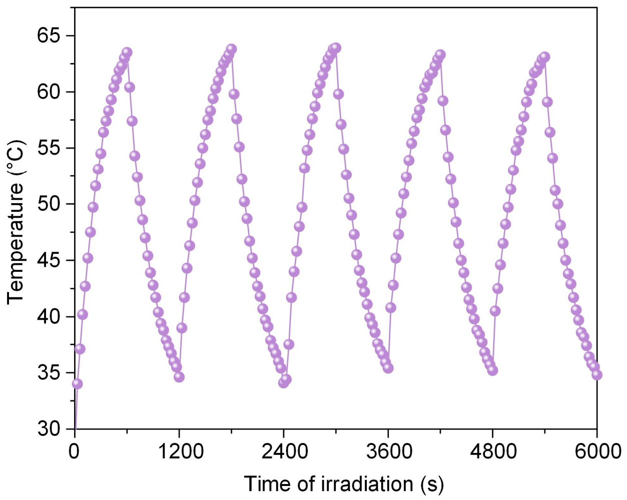

Figure S24. Photothermic stability of FeN_3P-SAC (100 μg mL^{-1}) upon 808 nm laser irradiation (10 min, 1.5 W cm^{-2}) for 5 on/off cycles.

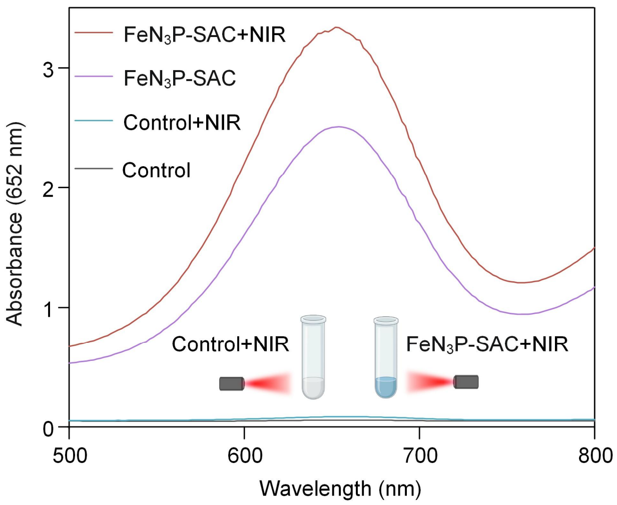

Figure S25. UV-Vis spectra for TMB oxidation after different treatments. The H_2O_2 and TMB concentrations were 1.462 mM and 0.623 mM, respectively. The FeN_3P-SAC concentration was 100 µg mL^{-1}. The control indicates no addition of samples.

Section S3. Supplementary Information Tables

Table S1. The EXAFS fitting structural parameters.

Sample	Shell	^{a}CN	^{b}R (Å)	$^{c}\sigma^2$ (Å^{-2})	$^{d}E_0$ *shift* (eV)	^{e}R factor
Fe foil	Fe–Fe	8	2.47(2)	0.005(2)	5.3(31)	0.007
	Fe–Fe	6	2.86(1)	0.005(3)		
FeN_3P-SAC	Fe–N	3.2(6)	2.01(1)	0.012(3)	0.1(21)	0.017
	Fe–P	0.7(1)	2.19(1)	0.020(5)		

^{a}CN: coordination numbers; ^{b}R: bond distance; $^{c}\sigma^2$: Debye-Waller factors (a measure of thermal and static disorder in absorber scatter distance); $^{d}E_0$ *shift* is edge-energy shift (the difference between the zero kinetic energy value of the sample and that of the theoretical model). ^{e}R *factor* is used to value the goodness of the fitting. The value of the amplitude reduction factor (S_0^2) lines between 0.7 and 1.

The *k*-range of 3 – 12 Å^{-1} and *R* range of 1 – 3 Å were used for the fitting of Fe foil.

The *k*-range of 3 – 10 Å^{-1} and *R* range of 1 – 3 Å were used for the fitting of samples of FeN_3P-SAC.

Section S4. Supplementary References

1. M. Gao, J. W. Fu, M. H. Wang, K. Wang, S. M. Wang, Z. W. Wang, Z. M. Chen and Q. Xu, *J. colloid interface Sci.,* 2018, **524**, 165–176.
2. J. Guo, B. L. Tardy, A. J. Christofferson, Y. Dai, J. J. Richardson, W. Zhu, M. Hu, Y. Ju, J. Cui, R. R. Dagastine, I. Yarovsky and F. Caruso, *Nat. Nanotechnol.,* 2016, **11**, 1105–1111.
3. Y. Han, K. Ge, Y. Zhao, M. Bottini, D. H. Fan, W. C. Wu, L. W. Li, F. S. Liu, S. T. Gao, X. J. Liang and J. C. Zhang, *Small,* 2024, **20**, 2306656.
4. D. Xi, M. Xiao, J. Cao, L. Zhao, N. Xu, S. Long, J. Fan, K. Shao, W. Sun, X. Yan and X. Peng, *Adv. Mater.,* 2020, **32**, 1907855.
